# The role of miR-200b/c in balancing EMT and proliferation revealed by an activity reporter

**DOI:** 10.1038/s41388-021-01708-6

**Published:** 2021-03-02

**Authors:** Paradesi Naidu Gollavilli, Beatrice Parma, Aarif Siddiqui, Hai Yang, Vignesh Ramesh, Francesca Napoli, Annemarie Schwab, Ramakrishnan Natesan, Dirk Mielenz, Irfan Ahmed Asangani, Thomas Brabletz, Christian Pilarsky, Paolo Ceppi

**Affiliations:** 1 Interdisciplinary Center for Clinical Research (IZKF), Friedrich-Alexander University of Erlangen-Nuremberg (FAU), Erlangen, Germany; 2 Department of Biochemistry and Molecular Biology, University of Southern Denmark, Odense, Denmark; 3 Department of Surgery, Friedrich-Alexander University of Erlangen- Nuremberg (FAU) and University Hospital of Erlangen, Erlangen, Germany; 4 Department of Cancer Biology, Perelman School of Medicine, University of Pennsylvania, Philadelphia, USA; 5 Department of Molecular Immunology, Friedrich-Alexander University of Erlangen-Nuremberg, Erlangen, Germany; 6 Department of Experimental Medicine-I, Friedrich-Alexander University of Erlangen-Nuremberg, Erlangen, Germany; 7 Present Address: Department of Oncology at San Luigi Hospital, University of Turin, Turin, Italy

**Keywords:** Cancer, Cell growth

## Abstract

Since their discovery, microRNAs (miRNAs) have been widely studied in almost every aspect of biology and medicine, leading to the identification of important gene regulation circuits and cellular mechanisms. However, investigations are generally focused on the analysis of their downstream targets and biological functions in overexpression and knockdown approaches, while miRNAs endogenous levels and activity remain poorly understood. Here, we used the cellular plasticity-regulating process of epithelial-to-mesenchymal transition (EMT) as a model to show the efficacy of a fluorescent sensor to separate cells with distinct EMT signatures, based on miR-200b/c activity. The system was further combined with a CRISPR-Cas9 screening platform to unbiasedly identify miR-200b/c upstream regulating genes. The sensor allows to infer miRNAs fundamental biological properties, as profiling of sorted cells indicated miR-200b/c as a molecular switch between EMT differentiation and proliferation, and suggested a role for metabolic enzymes in miR-200/EMT regulation. Analysis of miRNAs endogenous levels and activity for in vitro and in vivo applications could lead to a better understanding of their biological role in physiology and disease.

## Introduction

MicroRNA (miRNA)s are short, highly conserved, RNAs that repress the expression of their target genes [1]. They directly/indirectly regulate many cellular processes like cell cycle, apoptosis, senescence, aging, and migration [2, 3], and have a role in both physiology and pathology. In cancer, miRNAs can act as tumor suppressors or oncomiRs [4–6], and have been linked with all hallmarks [6–11]. Epithelial-to-mesenchymal transition (EMT) is an embryonic development program, which many cancers hijack to increase the migratory and invasive capacities [12]. EMT is enforced by specialized transcription factor families like SNAIL, basic helix loop helix (bHLH), TWIST, and ZEB [13, 14], which can work as E-Cadherin repressors and promote the expression of EMT effector genes. MiRNAs can target EMT-inducing transcription factors [13, 15]. For example, miR-200 inhibits the transcription factors of the ZEB family in a reciprocal feedback loop [16] and miR-34 can target SNAIL [17]. The equilibrium between EMT-suppressing miRNAs and key EMT transcription factors determines the epithelial cellular plasticity, see Supplementary Fig. 1A.

MiRNAs are usually functionally investigated by overexpression or knocking-down techniques, and the downstream effects are measured by biochemical identification of target genes and by the analysis of cellular phenotypes. However, high-throughput functional assessment indicated that the majority of miRNAs expressed in cells do not show detectable targeting activity [18], suggesting the need for robust miRNA functional reporters to better investigate their physiological role. MiRNA sensors have been recently developed [18–21], and in limited cases used to isolate cells with distinct biological properties [22]. However, the systems so far proposed were based on artificial 3′UTRs containing multiple repeated perfect complementarity sequences or miRNA binding sites, instead of naturally occurring 3′UTRs, and generally lacked essential non-binding mutant controls [23].

**Fig. 1:**
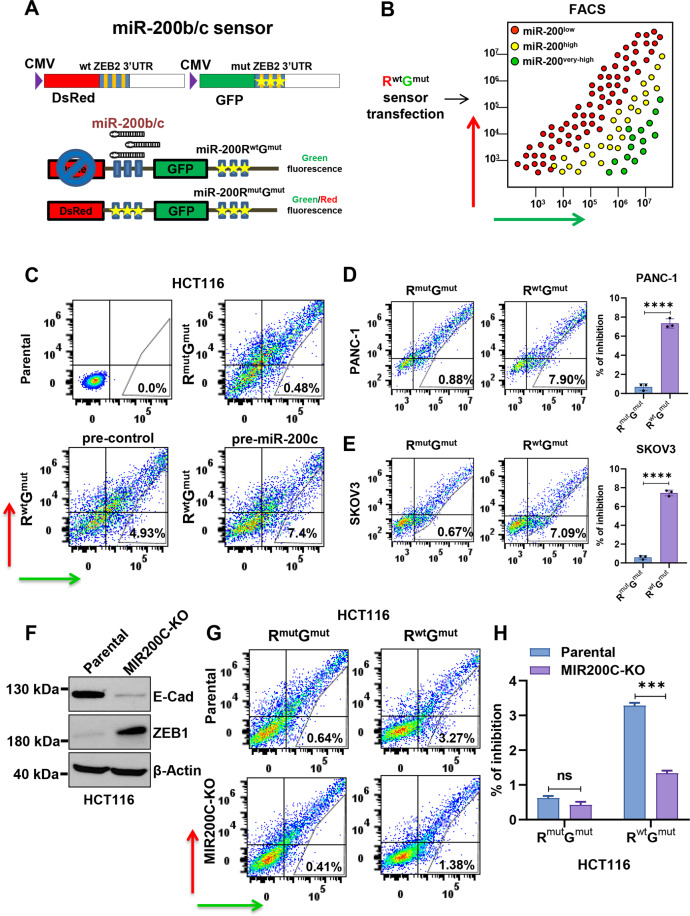
The miRNA sensor plasmid can be transiently transfected in cells to detect miR-200b/c levels and distinguish differential EMT states. **A** Schematic representation of the miR-200b/c sensor. GFP Green Fluorescent Protein, DsRed Discosoma Red Fluorescent Protein, R^mut^G^mut^ and R^wt^G^mut^. wt wild-type, mut mutated, ZEB2 3′UTR 3′ untranslated region of ZEB2 gene. **B** Schematic representation of the separation of miR-200b/c sensor-transfected cells in a FACS plot. The *X* axis represents green fluorescence intensity, and the *Y* axis represents red fluorescence intensity. Red dots indicate miR-200b/c low cells, yellow dots are miR-200b/c high cells, and in green are cells with very high miR-200b/c levels (for instance of exogenous source). **C** FACS plots showing the fluorescence intensity of HCT116 cells with FITC-A (Green) and PE-A (Red) channels after transfection with sensor plasmid in the presence of either pre-control or pre-miR-200c at 100 nM concentration. Indicated are the percent of gated cells over the total amount of cells in the experiment, including un-transfected. **D** FACS plots showing transfection of PANC-1 cells with R^mut^G^mut^ or R^wt^G^mut^ plasmids and bar graphs showing the percent of inhibition. **E** FACS plots showing transfection of SKOV3 cells with R^mut^G^mut^ or R^wt^G^mut^ plasmids and bar graphs showing the percent of inhibition. **F** Western blot quantification of ZEB1 and E-Cadherin protein expression of HCT116 cells with miR-200c knockout (MIR200C-KO), compared to parental cells. β-Actin was used as a loading control. **G** FACS plots showing the transfection of sensor plasmids in MIR200C-KO HCT116 cells or in parental control cells. **H** Bar graphs showing the percent of inhibition of FACS analysis done in (**G**). In **D**–**E** *p* values are from Student’s *t* test. In **H** *p* values are from two-way ANOVA. Points are average ± SD. *<0.05, **<0.01, ***<0.001, ****<0.0001.

To develop a robust and controlled plasmid-based fluorescent miRNA sensor, we cloned a 3′UTR fragment of the miR-200 target ZEB2 [14], containing three strong miR-200 seed matches fused to DsRed (R) fluorescent protein, driven by a CMV promoter. As a non-targeting control, in the same plasmid we fused GFP (G) to the ZEB2 3′UTR carrying mutated seed matches, driven by an identical CMV promoter (the R^wt^G^mut^ vector). Once expressed in living cells, this plasmid is designed to respond to miR-200 levels altering the intensity of the red fluorescence, while green fluorescence serves as a transfection control. A double mutant non-binding control vector (R^mut^G^mut^) was also generated, Fig. 1A. We previously showed that the R^wt^G^mut^ sensor is highly specific for the miR-200 b/c/429 cluster [24]. In this study, we report that EMT biology can be explored in living cells by its transient transfection, and this approach could be exported to virtually all biologically relevant miRNAs in different cellular models, with several important applications for miRNA research.

## Results

### The sensor plasmid can detect endogenous miR-200b/c activity

The dual fluorescence sensor was overexpressed in cells by transient transfection and its ability to report endogenous miR-200b/c levels was monitored by FACS. The scheme in Fig. 1B illustrates the different cell populations based on their miR-200b/c levels, as visualized in a FACS following sensor transfection. As a cellular model, we chose the colorectal cancer (CRC) cell line HCT116, which expresses high levels of miR-200s [14] and has an epithelial-like phenotype [25]. HCT116 cells were co-transfected with R^mut^G^mut^ control plasmid in the presence of miR-200c mimics (pre-miR-200c) or scrambled control (pre-control), and the green and red fluorescence intensity were recorded on a flow cytometer. As a result, we found that the double mutant plasmid allowed a robust and coordinated expression of the fluorescent transgenes (cells along a diagonal line in the FACS plot), an important pre-requisite for the detection of changes of signal intensities due to the binding of endogenous miRNAs. Overexpression of pre-miR-200c produced no detectable alteration of fluorescence in these cells, Supplementary Fig. 1B, indicating the lack of binding activity. By contrast, using the signal from the non-binding R^mut^G^mut^ plasmid to set the FACS gates, we could observe the appearance of a population with reduced red intensity in cells transfected with the R^wt^G^mut^ sensor (with scrambled control), Fig. 1C. This result suggests that, when expressed in cells with detectable levels, the miR-200b/c sensor can report a significant binding activity in a sub-population of cells, potentially carrying the highest endogenous miR-200b/c levels. The percentage of cells in the gated area for high miR-200b/c further increased upon miR-200c overexpression, Fig. 1C, suggesting the notion that the sensor can be used to monitor cells with high miR-200b/c from endogenous and exogenous source. Similar results were obtained from another miR-200b/c positive cell line, the human bladder carcinoma RT112, Supplementary Fig. 1C, and endogenous levels were visualized also in other cancer cell lines, like PANC-1 and SKOV3, Fig. 1D, E.

### The sensor reports miR-200 activity in cells with differential EMT states

The miR-200 family has a strong EMT-repressing role and is normally reduced in mesenchymal-like cells [15]. We sought to determine the activity of the sensor in cells with altered EMT status using an isogenic cellular system with high and low miR-200. For this, we tested mouse pancreatic cancer cell lines derived from a Pdx1-cre;Kras^LSL.G12D/+^;Tp53^LSL.R172H/+^ mouse model (KPC), which has been further modified to obtain the Zeb1 knockout, called KPCZ, to show a role of Zeb1 in the metastatic cascade of pancreatic tumors [26]. The mature sequence of miR-200b/c is highly conserved between human and mice [27]. Cells obtained from KPCZ tumors showed a more marked epithelial-like phenotype, Supplementary Fig. 2A, B, compared to KPC cells, with increased expression of miR-200 members b and c, Supplementary Fig. 2C. Upon transfection with miRNA sensors, KPCZ cells showed a superior inhibition rate, see Supplementary Fig. 2D, E, in line with the data obtained with the pre-miR transfection experiment.

To further test the specificity of the detection, we used a CRISPR/Cas9-based approach to permanently knockout miR-200c, the most prominent EMT-suppressing member of the miRNA family [28], taking advantage of a protospacer adjacent motif (PAM) sequence adjacent to the miRNA-200c seed match, Supplementary Fig. 3A. As a result, MIR200C-KO HCT116 cells showed a drastic reduction of miR-200c levels, as evaluated by qPCR, Supplementary Fig. 3B. In light of the high-sequence homology between the miR-200b and c, we also verified the effect of the KO on the MIR200B gene and a significant >2-fold reduction was also detected, Supplementary Fig. 3C. DNA sequencing on single-cell clones obtained from MIR200C-KO cells showed alterations around the PAM in the miR-200c, but not in the miR-200b region, Supplementary Fig. 3D, suggesting that the observed reduction in miR-200b is likely an indirect effect due to EMT induction, possibly mediated by ZEB1 increase. Phenotypic analysis of the MIR200C-KO cells, in fact, showed that they had undergone EMT, as indicated by a pronounced mesenchymal-like morphology and dispersed growth pattern, and confirmed by western blotting and immunofluorescence staining (Fig. 1F and Supplementary Fig. 4A). To check that EMT was propelled by the miR-200c knockout and not by an unwanted off-target effect [29], we reconstituted the cells with exogenous miR-200c by transient transfection and could observe an attenuation of the phenotype, Supplementary Fig. 4B. Once transfected with miR-200b/c sensors, the miR-200C-KO cells displayed a significant reduction in the percentage of cells with inhibition of red fluorescence, Fig. 1G, H, further supporting the high specificity of the miR-200b/c sensor system. Overall, these data indicated that the sensor can monitor changes in the miR-200b/c levels in living cells with distinct EMT states.

### Utility of the sensor to sort cells by endogenous miR-200b/c levels/EMT status

**Fig. 2:**
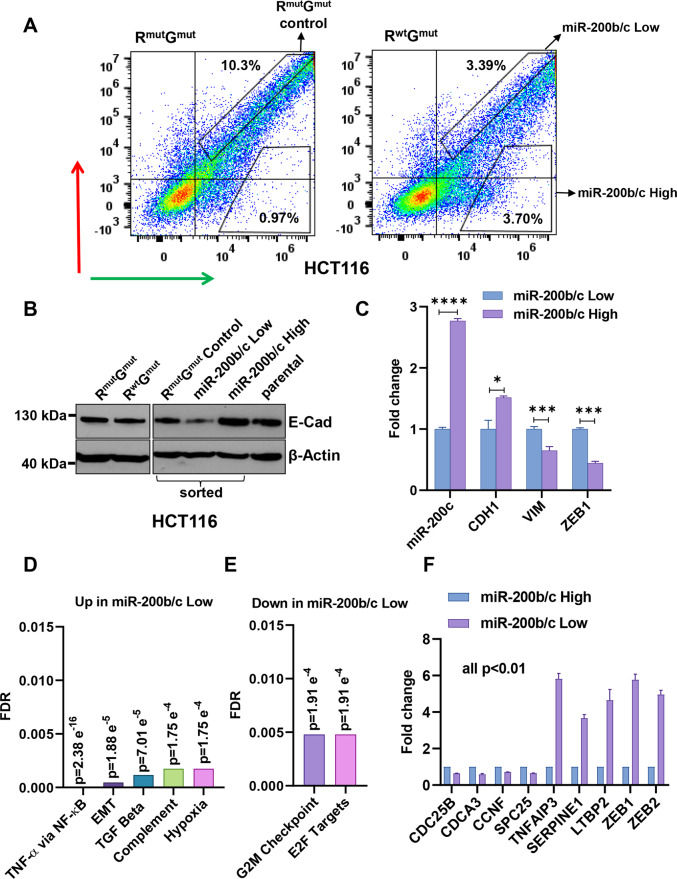
The sensor can be used to sort cells by endogenous miR-200b/c levels. **A** FACS plots showing the gates used for sensor-sorting miR-200b/c low and high cells after transfection with the R^wt^G^mut^ plasmid (right panel). Left panel shows R^mut^G^mut^ transfected control cells and their gate for sorting control. **B** Western blot quantification of E-Cadherin in HCT116 cells transfected either with R^mut^G^mut^ or R^wt^G^mut^ plasmids (unsorted, left panel), or sorted with the gates shown in (**A**) and re-plated for 3 days to grow and remove dead cells (right panel). **C** qPCR quantification of relative mRNA levels of CDH1 (gene coding E-Cadherin), VIM (Vimentin), ZEB1 and miR-200c in miR-200b/c low or high HCT116 cells sorted as in (**A**). Gene set enrichment analysis of RNA-sequencing data depicting pathways **D** upregulated and **E** downregulated in sensor-sorted miR-200b/c low cells compared to miR-200b/c high cells. **F** qPCR validation of RNA-sequencing data. CDC25B, CDCA3, CCNF, SPC25, TNFAIP3, SERPINE1, LTBP2, ZEB1 and ZEB2 were quantified using GAPDH as a housekeeping gene. *p* values are from Student’s *t* test. Points are average ± SD. *<0.05, **<0.01, ***<0.001, ****<0.0001.

We then tested the possibility to use the sensor for FACS-sorting cells with a differential EMT status based on the endogenous high- and low-miR-200b/c expression. HCT116 cells were transfected with R^wt^G^mut^ and R^mut^G^mut^ plasmids on a larger scale and separated by their R^wt^G^mut^ non-inhibited (miR-200b/c low) and red-inhibited (miR-200b/c high) population, Fig. 2A. Gated areas were selected to collect cells with the same intensity of green fluorescence (the mutant control) and a significant difference in red. After sorting, cells were re-plated and allowed to attach and grow for 3 days to recover, to exclude dead cells from the further characterizations and to increase in number. A western blot conducted to monitor the expression of the epithelial marker E-Cadherin identified no change in E-Cadherin abundance in R^wt^G^mut^ compared to R^mut^G^mut^ transfected cells (unsorted), to control that the miR-200b/c sensor itself did not alter the EMT phenotype. Additional experiments on HCT116, RT112, and PANC-1 parental unsorted cells were further performed to better rule out the possibility that the sensor was sequestering miR-200b/c and favouring EMT upon transfection, Supplementary Fig. 5A, B. Analysis of sorted cells showed that E-Cadherin levels were also unaltered in R^mut^G^mut^ compared to parental cells, while a significant reduction was found in miR-200b/c low, and a relatively marked increase was detected in miR-200b/c high cells obtained from R^wt^G^mut^ transfection, Fig. 2B. In a separate experiment, qPCR analysis confirmed the differential gene expression of miR-200c and EMT markers in miR-200b/c high- and low-sorted cells (Fig. 2C). Similar results were obtained by sorting sensor-transfected RT112 cells, Supplementary Fig. 5C, D. In addition, we found that sorted high and low cells had a strong propensity for quickly reverting their fluorescence after re-plating, as quantified by FACS analysis and video imaging (Supplementary Fig. 5E, F), indicating that miRNA activity can be monitored for a few days in vitro in individual sensor-transfected cells. Interestingly, despite the fact that a differential EMT status could not be evidenced by the microscopic morphological examination of sorted cells, miR-200b/c low clearly showed a significantly lower ability to attach after sorting, compared to high cells. Quantifications confirmed that they were more rounded and of smaller size when re-plated, indicating a lower adhesion capacity, before normalizing in the following days (Supplementary Fig. 5G, H). Altogether, this was the first indication that the sensor-guided FACS-sorting strategy was capable of physically separating cells with distinct miR-200b/c levels and EMT properties.

### miR-200b/c sensor-sorted cells can be used to molecularly characterize endogenous EMT states

EMT is a complex multifactorial process. Although a few markers like E-Cadherin, Vimentin and ZEB1 can represent a good surrogate for the determination of the EMT state [14, 30], this should be better concluded from broader, and possibly genome-wide characterizations [31]. We, therefore, repeated the sorting experiment twice independently (with duplicates) and subjected all the eight RNAs isolated from high and low cells to sequencing. Using a cut-off of twofold for defining differentially expressed genes, we identified 224 upregulated and 73 downregulated genes in the miR-200b/c low compared to high cells, as an overlap between the two distinct sorting experiments, see Supplementary Fig. 6A and Supplementary Table 1A, B. A geneset enrichment analysis revealed that the signatures most significantly upregulated in the miR-200b/c low cells were belonging to the TNF-alpha signaling via NF-κB, to EMT, and the TGF-beta signaling pathways, Fig. 2D. On the other hand, analysis of the genes downregulated in miR-200b/c low cells indicated the significant prevalence of cell-cycle-related targets, like those linked with the E2F transcription factors and genes involved in the G2/M progression through the cells division cycle, Fig. 2E. Of note, ZEB1 and ZEB2 were identified among the most differentially expressed genes, Supplementary Fig. 6B. This is particularly important as a further assay validation, since the sensor plasmid is designed as a functional readout of the miR-200b/c binding to the ZEB transcription factors. Moreover, clustering analysis of miR-sensor low and high cells with the parental HCT116 cells indicated that HCT116 cells resembled and clustered with miR-200b/c high cells, considering both differentially expressed genes (Supplementary Fig. 6C) and known miR-200b/c target genes (Supplementary Fig. 6D-F), in line with the fact that HCT116 have a predominant epithelial-like nature. Quantitative PCR was used to independently validate the RNA-sequencing results and confirmed the down- or upregulation of relevant genes in the identified pathways, like CDCA3 among the E2F target genes and TNFAIP3 for TNF-alpha signaling along with ZEB1 and ZEB2, Fig. 2F.

**Fig. 3:**
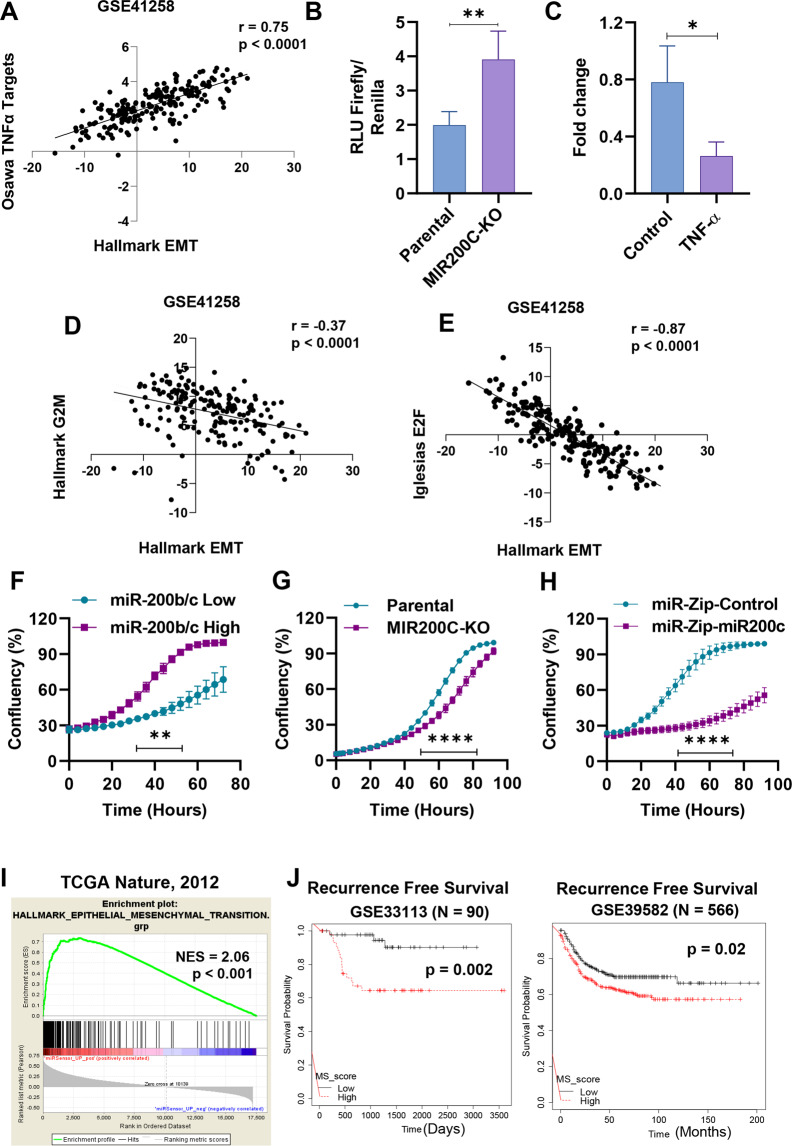
miR-200b/c sensor-sorted cells can be used to molecularly characterize endogenous EMT states. **A** Correlation between the *z*-scores of hallmark EMT and Osawa TNF-α targets in GEO dataset GSE41258. **B** Bar graphs showing the relative NF-κB activity (RLU Firefly/Renilla reporter assay) in parental and miR-200c KO (MIR200C-KO) HCT116 cells. **C** qPCR quantification of miR-200c levels in HCT116 cells after treatment with 30 ng/mL TNF-α. **D** Correlation between the *z*-scores of Hallmark EMT and Hallmark G2M targets in GSE41258. **E** Correlation between the *z*-scores of Hallmark EMT and Iglesias E2F targets in GSE41258. Real-time proliferation quantification in (**F**) FACS-sorted miR-200b/c high- and low-HCT116 cells, **G** Parental and MIR200C-KO HCT116 cells, and **H** HCT116 cells stably infected with either miR-Zip control or miR-Zip-200c. **I** Gene-set enrichment analysis of genes upregulated in miR-200b/c low cells with hallmark EMT gene-set in the indicated dataset using Pearson’s gene metric. **J** Kaplan–Meier analysis of recurrence-free survival in colorectal cancer patients (GSE33113 and GSE39582) based on the median value of MS score. *p* values in (**J**) are calculated using log-rank test. In **B** and **C** the *p* values are from Student’s *t* test. In **F**–**H** *p* values are from two-way ANOVA and Sidak’s multiple test. Points are average ± SD. *<0.05, **<0.01, ***<0.001, ****<0.0001.

We next sought to functionally validate the role on EMT of the pathways associated with the gene signatures obtained from sensor-sorted cells. TGF-beta is an established master EMT inducer [32]. TNF-alpha has also been reported as an EMT-inducing cytokine in different cancer cell lines, including cells from colorectal origin, with and without the NF-κB-mediated upregulation of EMT transcription factors [33–35]. Importantly, we were able to verify a strongly significant association between TNF-alpha and EMT gene signatures in colorectal cancer patients, Fig. 3A and Supplementary Fig. 6G, indicating the clinical relevance of the signatures obtained from sensor-sorted cells. To validate these findings in our cellular system, we measured NF-κB activity by a luciferase reporter assay in MIR200C-KO cells and found it elevated, Fig. 3B. Moreover, TNF-alpha treatmentinduced EMT markers in parental HCT116 cells, Supplementary Fig. 6H, and significantly reduced miR-200c expression levels, Fig. 3C. These results confirmed that TNF-alpha and NF-κB pathways can influence EMT and miR-200 in HCT116 cells, as well as the robustness of the data generated with the sensor-sorting strategy. Next, we also aimed to validate the proliferative signature obtained from the miR-200b/c high cells. Supporting evidence came from a few pivotal studies showing that miR-200b/c can sustain CRC growth [36, 37], and strong inverse correlations was confirmed between G2M and E2F gene signatures and EMT in CRC patients, Fig. 3D, E. As a functional assay, we measured the growth of the sorted miR-200b/c high and low cells using an automated real-time imaging system to monitor cellular confluency. The results indicated that the high cells grew significantly faster compared to low cells, Fig. 3F and Supplementary Fig. 7A. On the other hand, MIR200C-KO HCT116 cells grew slower than parental cells, Fig. 3G. Moreover, shRNA-mediated knockdown of miR-200b/c (using lentiviral miR-Zip technology) was carried out and independently confirmed that HCT116 cells were strongly dependent on miR-200 for their growth, Fig. 3H. This observation was replicated in another CRC cell line COLO205 (Supplementary Fig. 7B). We then reasoned that, because of their increased proliferative status, miR-200b/c high cells could have been more sensitive to cell-cycle inhibition. We therefore subjected HCT116 cells to treatment with a potent inhibitor of a cyclin-dependent kinase, CGP-60474, in the presence or absence of the pre-miR-200c. The results indicated that pre-miR-200c transfected cells had a significantly higher growth reduction with CGP-60474, compared to control cells, Supplementary Fig. 7C, D. In line with this, CGP-60474 induced a dose-dependent downregulation of miR-200b/c high cells in sensor-transfected HCT116, Supplementary Fig. 7E, F. Altogether, this functional validation corroborated the notion that the miR-200b/c sensor can represent a valuable tool to separate and characterize cells based on their functional EMT states and can be used to discover biological properties related to miRNA activity.

Finally, we performed a geneset enrichment analysis of genes upregulated in miR-200b/c low cells with hallmark EMT gene-set and found very significant association in three independent gene datasets, Fig. 3I and Supplementary Fig. 8A, confirming the clinical significance of the data obtained from the sorting experiment. Moreover, when prognostically evaluated, high expression of the gene signature showed to predict poor recurrence-free survival in CRC patients, Fig. 3J and Supplementary Fig. 8B. Therefore, the gene signatures obtained from miRNA sensor-sorted cells correlated with clinical EMT and proved to be relevant for prognostic studies. Finally, to further validate the relevance of the method to explore miRNA functions, we tested if the associations identified with the sensor-based approach could have been predicted by an expression analysis, for instance by dividing CRC patients in two groups based on miR-200c expression. As a result, we found that this more standard approach failed to significantly identify TNF-alpha and G2M/E2F signatures as enriched in patients with low and high miR-200c, respectively, Supplementary Fig. 9, further indicating that the analysis of miRNA activity at the endogenous level we deployed can be very effective for obtaining biologically and clinically relevant information and study miRNA functions.

### The sensor can be used to identify miR-200b/c upstream regulators

**Fig. 4:**
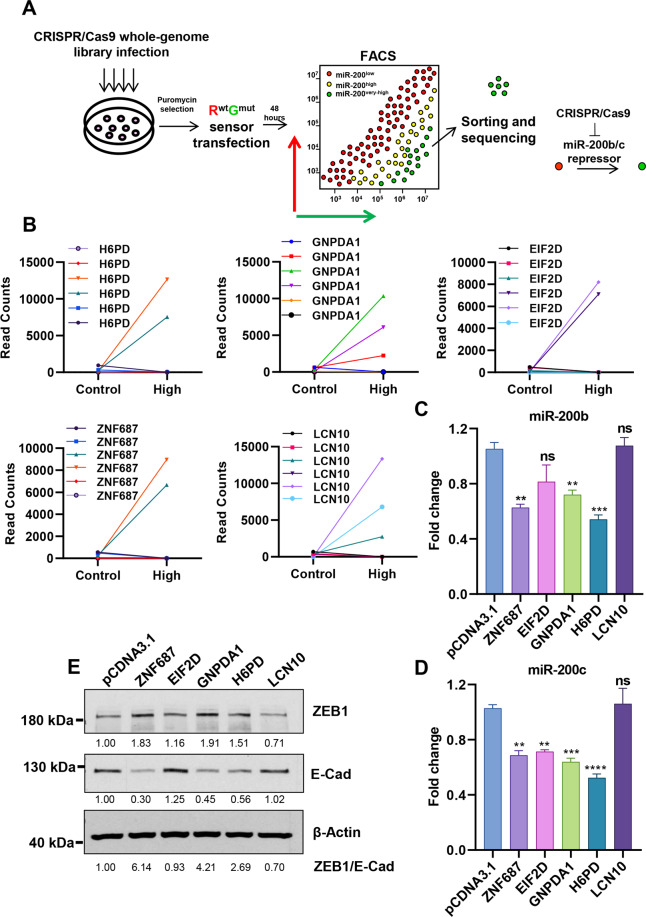
The sensor can be used to identify miR-200b/c upstream regulators . **A** Scheme showing the CRISPR-Cas9 screening strategy to identify miR-200b/c regulators using the miR-200b/c sensor. **B** Graphs showing read counts for the indicated genes’ gRNAs between sorted miR-200b/c high and control cells. Validation of the top hits from the CRISPR/Cas9 screen by qPCR. miR-200b (**C**) and miR-200c (**D**) levels were quantified in cells stably transfected with the cDNA clones overexpressing the hits genes, compared to the pCDNA3.1 vector control. **E** Western blot quantification of EMT markers ZEB1 and E-Cadherin in cells as in (**C**, **D**). ZEB1 and E-Cadherin band intensities were calculated by densitometry. β-Actin was used as a loading control. *p* values are from Student’s *t*-test. Points are average ± SD. *<0.05, **<0.01, ***<0.001, ****<0.0001.

As we established that the miRNA sensor functions as an effective fluorescence reporter, we tested the possibility of using it to identify the upstream regulators of miR-200b/c. To do so in an unbiased and genome-wide fashion, we deployed a whole-genome CRISPR-Cas9 library platform and combined it with the sensor/sorting strategy. HCT116 cells were first lentivirally infected to overexpress the Cas9 nuclease and then with the virus obtained from the pooled CRISPR-Cas9 library [38]. Around 3 × 10^8^ HCT116-Cas9-library cells were sensor-transfected and FACS sorted in multiple rounds based on their miR-200b/c levels. MiR-200b/c very high population was expected to be enriched with cells carrying the knockout of genes with the ability to directly/indirectly repress miR-200b/c, Fig. 4A. After sorting, cells were expanded, their DNA isolated, PCR amplified and sequenced (all gRNAs are barcoded). As a measure of library representation, a total of 107,197 gRNAs were detected in unsorted HCT116 cells expressing the CRISPR library (all gRNAs excluding those with no counts) about 90% of the total, in line with other screens that identified a similar amount of life-essential fitness genes [39]. In sorted miR-200b/c high cells, this number dropped to 18,817, due to the limited amount of cells that could be FACS-sorted, Supplementary Fig. 10. By comparing each gRNA abundance between control cells and miR-200b/c high cells, we identified a top-10 list of potential miR-200b/c repressors (Supplementary Table 2). The presence of two or more independent gRNAs with a significant enrichment in the miR-200b/c high cells was used as an additional statistical selection criteria (Fig. 4B). To functionally validate that an actual miR-200b/c repressor has been found, we took a functional approach and independently overexpressed the corresponding ORFs from five of the genes in HCT116 cells. Following generation of stable overexpressing cells, we quantified miR-200b and miR-200c expression by qPCR and found that three of them (H6PD, GNPDA1 and ZNF687) significantly reduced levels of both miRNA family members (Fig. 4C, D). Western blot quantification of EMT markers indicated that these genes had regulatory effect on EMT phenotype, and for two of them, H6PD and GNPDA1, stable overexpression was confirmed (Fig. 4E, and Supplementary Fig. 11A). Notably, the H6PD gene was also found significantly co-expressed with the EMT markers like ZEB1 and Vimentin in a CRC patients’ gene expression dataset, Supplementary Fig. 11B.

Conversely, to test if this technique was also able to identify genes with miR-200b/c-activating functions, we performed an independent screening sorting for the cells with low miR-200b/c (no inhibition of the red fluorescence). Among the top 5 identified (Supplementary Fig. 12A) we could validate by stable overexpression one gene, HSD17B1, as able to increase the E-Cadherin levels in HCT116 cells, and to upmodulate miR-200b and c (Supplementary Fig. 12B–D).

**Fig. 5:**
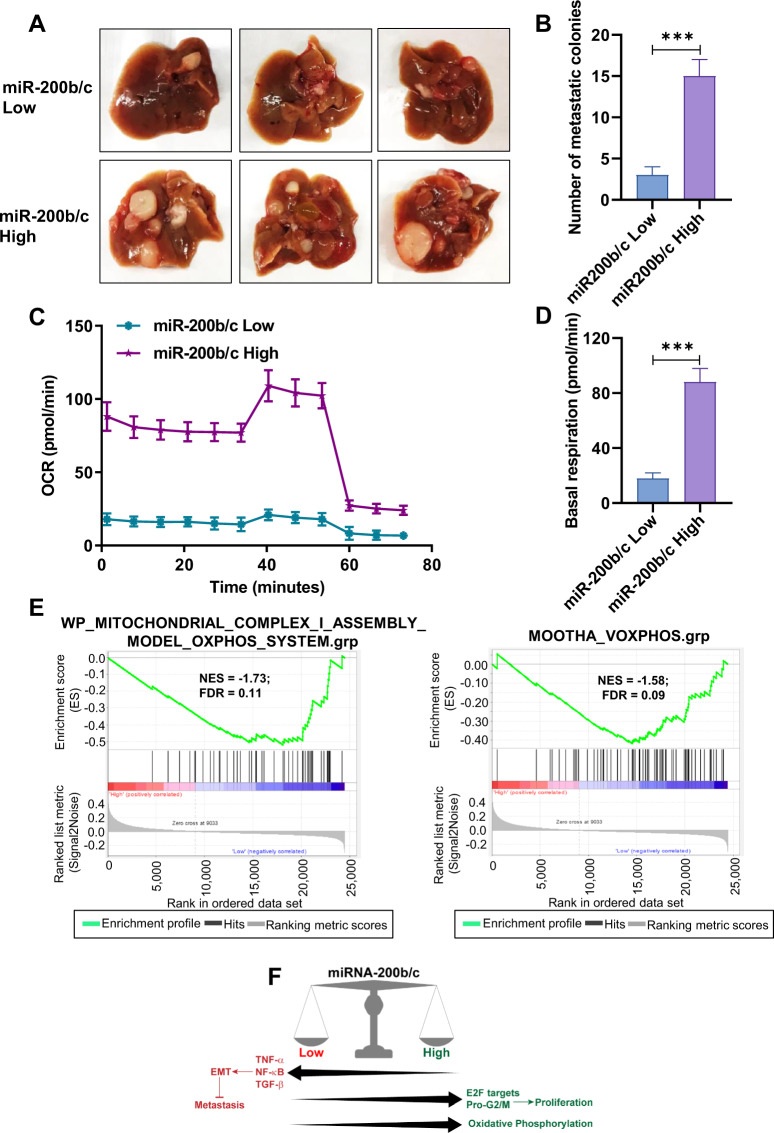
miR-200b/c high and low cells have different metastatic and metabolic properties. **A** Images of livers from NSG mice (*n* = 3) 5 weeks after injection of sorted HCT116 miR200b/c low or high cells, showing the metastatic colonies. **B** Bar graph showing the average metastatic colonies from (**A**) ±SD. **C** Oxygen Consumption Rate (OCR) measured by seahorse flux analyzer in HCT116 miR-200b/c low or high cells. **D** Bar graph showing basal respiration in HCT116 miR-200b/c low or high cells from (**C**). **E** Gene-set enrichment analysis of OXPHOS related genesets with the low and high categorized patient samples based on miR-200b/c sensor RNA-seq genes expression in the gene expression dataset GSE81980. GSEA was performed with the ranking of genes with signal2noise metric. **F** Scheme of the identified role of miR-200b/c as a molecular switch between EMT and proliferation. In **B** and **D** *p* values are from Student’s *t* test. Points are average ± SD. *<0.05, **<0.01, ***<0.001, ****<0.0001.

Finally, to assess the in vivo relevance of the miRNA sensor-sorting strategy, we performed a metastasis assay on HCT116 cells transfected and sorted for low and high miR-200b/c, manually re-counted and directly injected in the tail vein of NSG mice. The results clearly indicated the ability of the sensor to discriminate cells for their metastatic potential, with the miR-200b/c low cells forming a significantly lower number of liver metastasis, Fig. 5A, B. This is in line with the partial EMT phenotype, recently identified to explain how terminally EMT-differentiated cells lose metastatic potential [40, 41], here shown also for a CRC cancer cell line.

Furthermore, we conducted a Seahorse metabolic analysis, which showed a drastic reduction of OXPHOS ability in the sorted miR-200b/c low cells, compared to high cells (Fig. 5C, D). This experimental observation was also independently validated at the computational level, as OXPHOS genes were found accordingly enriched in the RNA-sequencing data from sorted cells (Fig. 5E), altogether providing a mechanistic metabolic explanation for the loss of growth observed in miR-200b/c low cells.

## Discussion

We report proof-of-concept demonstration that miRNA sensors with natural 3′UTRs and non-binding control sequences can work as functional reporters to detect miRNA expression/activity. They permit the physical separation and the in vitro and in vivo characterization of cells with differential baseline miRNA expression levels, and can be combined with high-throughput functional screening technologies. Since miRNAs are key regulators of physiological and pathological mechanisms [42], this system might have important implications in miRNA research and can be optimized to be used in several applications, like miRNA target identification studies or for real-time tracing miRNA activity in single cells in vitro. In the case of miR-200b/c, for instance, the latter feature could be used to visualize the high degree of cellular plasticity normally seen in EMT states [43], as well as in pharmacological studies to screen drugs targeting the EMT process [44]. Nevertheless, the sensor can also be used to infer fundamental biological properties of miRNAs. In terms of ability to distinguish endogenous levels, the RNA profiling results here reported are very encouraging, as the cells with low and high endogenous miR-200b/c matched the corresponding expression of EMT markers and the most important elements (like the pro-proliferative role) were independently functionally validated. Of note, the biological associations identified by the sensor could not have been predicted with an expression-based analysis, confirming the significance of this approach to investigate miRNA biology. RNA-sequencing on sensor-sorted cells indicated a strong upregulation of EMT, the TNF-alpha and the TGF-beta signaling pathways in cells with low miR-200b/c. Both the TNF-alpha and the TGF-beta signaling pathways have been shown to determine the EMT phenotype of colorectal carcinomas [33, 45, 46], and can also synergistically converge and cooperate for EMT induction [47], as also revealed by our independent analyses. This implies that the miRNA sensor can be used to study complex molecular associations and identify crucial cell biological mechanisms. In our experimental model, the sensor revealed that miR-200b/c acts as key switch between EMT differentiation and proliferation, being miR-200b/c low cells featured with a strong EMT signature and high miR-200b/c cells more directed toward sustained proliferation, Fig. 5F. The dualism between EMT and proliferation has been previously suggested or proven in other cancer models [48], and some EMT factors have been found to actively repress proliferation, also in CRC [49, 50]. MiR-200 re-expression is known to induce tissue colonization via the reverse EMT process (MET) and the promotion of proliferation could facilitate the metastatic out growth [13]. MiR-200b/c could, therefore, be regarded as a powerful regulator of cellular fate, and deeper investigations on the underlying mechanisms may be crucial in understanding the heterogeneity and the adaptive plasticity frequently observed in tumors [51]. To further explore these critical aspects, the miR-200b/c sensor could be in the future combined with single-cell sequencing, contributing to reveal the molecular features of EMT intermediate states (referred to as partial or hybrid EMT), the fundamental importance of which has been recently clearly demonstrated in the metastatic process [40, 41, 52]. The new emerged model is also confirmed by our in vivo assay, in which cells with lower miR-200b/c levels and more complete EMT profile showed a reduced metastatic capacity.

Another important application is the unbiased analysis of miRNAs upstream regulation network. Regulation of miRNAs is primarily investigated by the analysis of their promoter regions (for the binding of specific transcription factors). Our pilot experiments using a whole-genome CRISPR/Cas9 library could identify a few unprecedented miR-200b/c regulators. The low number of hits is likely an effect of the great representation loss due to the sorting of a relatively low number of cells, which could be improved by increasing the sensitivity of the sensor (see below). However, we could functionally validate some genes, which, of note, belonged to ‘unexpected’ pathways, like metabolism. This confirms the power of this unbiased technique and points at a strong connection between EMT and cancer metabolism, which is a very lively field of investigation with several important translational implications [53]. H6PD is an endoplasmic reticulum specific enzyme that functions as a glucose 6-phosphate dehydrogenase and converts it to 6-phospho glucano lactone. GNPDA1 converts glucosamine-6-phosphate to fructose-6-phosphate and vice versa. These two glucose metabolism enzymes work closely associated (Supplementary Fig. 13A) and could be further explored in EMT regulation. This is not completely surprising, as the connection between EMT and glucose metabolism has recently started to emerge in the literature [30, 54]. Remarkably, also the miR-200b/c activator gene here identified, HSD17B1, is a metabolism gene, being part of the testosterone/estrogen synthesizing pathway (Supplementary Fig. 13B). Estrogen signaling has been previously associated with EMT suppression in CRC via the upregulation of another EMT-repressing miRNA, miR-205 [55], which can work in association with miR-200 [11] and, along the same line, androgen-deprivation therapy has been shown to induce EMT via ZEB1 [56]. In line with the possible strong role of metabolism in regulating the fate of cellular growth and differentiation, also mediated via miR-200b/c, are the data from our Seahorse profiling experiment, which provided a mechanistic rationale for the phenotypes observed in the sorted cells. In conclusion, even if these findings will require further experimental verifications, addressed for instance to understand the direct/indirect nature of the regulation and the exact metabolic players involved, they highlight the great potentiality of the technique. Future screens performed in larger settings and with improved miRNA sensors (as described below) will allow high-resolution functional mapping of the regulatory events upstream of miRNAs.

All the above mentioned possible applications, extended to virtually all miRNAs, will be facilitated by a significant further development of the technique here presented. The miR-200b/c sensor design enables a high level of specificity [24, 57], here confirmed by the analysis of endogenous levels in different cellular models and by overexpression and knockdown approaches. However, the sensitivity can be the object of further improvements, allowing to extend the assay to cells with lower miRNA endogenous expression. Sensors designed with artificial 3′UTRs with multiple fragments perfect complementary to the targeting miRNA can guarantee a higher level of sensitivity, as previously shown [58]. However, the sensor here presented has the advantage of carrying naturally occurring sequences properly controlled with non-binding mutants, in analogy with the dual reporter vectors used to validate bona fide miRNA targets. This method is preferable to better represent physiological conditions [58] by (1) making sure that the sensors are reliably reporting endogenous miRNA activity, i.e., not overestimating their effects, and by (2) minimizing or eliminating miRNA-sponge or decoy effects [18, 59], which could alter the biological properties. Another unappreciated factor is that the presence of non-natural 3′UTRs (either containing repeated seed matches or complementary sequences) carrying random spacer sequences increases the chances to introduce novel unwanted, albeit specific, miRNA binding sites. In the absence of non-binding controls, the contribution of these off-target detections can be difficult to estimate. To improve sensitivity, therefore, multiple 3′UTRs controlled with non-binding mutants in a 1:1 ratio could in future be cloned in tandem and tested. Another limiting factor is that this sensor can only be transiently introduced in the cells, thus limiting the number of cell lines to be potentially investigated and the amount of output cells per sorting round, an important factor for the downstream techniques requiring higher amount of cells. MiRNA-reporter cells with integrated sensors would allow better live tracking of miRNAs activity in experimental settings in vitro and in vivo. However, integration of this vector into the genome produced recombination events possibly due to the highly repetitive sequences [24], a fact that precluded its further use. To overcome this limitation, future studies should be conducted to improve the sensor architecture, like using single bidirectional promoter vectors [60] to minimize the presence of repetitive sequences. Once improved, this approach could be implemented for all biologically relevant miRNAs, leading to fundamental discoveries in biomedicine.

## Methods

### Cell culture and chemicals

HCT116 cells were cultured in McCoy’s 5A (Lonza), RT112 and COLO205 cells were cultured in RPMI1640 (Sigma), PANC-1 and HEK 293 (all from ATCC) were cultured in DMEM (Sigma), and SKOV3 cells (NCI) were cultured in RPMI1640 supplemented with 1 mM sodium pyruvate (Gibco), 1% MEM NEAA (Gibco), and 1% MEM Vitamin solution (Gibco). Media were supplemented with 10% FBS (Sigma), 1% pencillin/streptomycin (Sigma) and 1% l-glutamine (Sigma). Cells were cultured at 37 °C and 5% CO_2_ in a humidified incubator. Cells were STR authenticated and tested for mycoplasma regularly (Invivogen). HCT116-Cas9 cell line was generated by lentiviral transduction with lentiCas9-Blast (Addgene) and selection with 4 μg/mL of blasticidin (Sigma) for 3 days. KPC (Pdx1-cre;Kras^LSL.G12D/+^;Tp53^LSL.R172H/+^) and KPCZ (Pdx1-cre;Kras^LSL.G12D^/+;Tp53^LSL.R172H/+^; ZEB^fl/fl^) are mouse pancreatic cancer cells without and with ZEB1 knockout [26] and were cultured in DMEM. TNF-alpha was from Gibco, and Cdk inhibitor (CGP-60474) from Tocris.

### FACS analysis

HCT116 cells were seeded in six-well plates at a density of 0.5 million per well. The next day cells were transfected with 1.5 µg of either R^mut^G^mut^ or R^wt^G^mut^ plasmids. After 48 h, cells were trypsinized, washed, and resuspended in FACS buffer (5 mM EDTA and 2% FBS/PBS). Samples were run on Cytoflex FACS machine (Beckman). FACS data were analyzed using FlowJo software v10.6.

### CRISPR screen

HCT116-Cas9 cells were transduced with lentiviral Human GeCKO v2 library part A and part B at MOI of 0.3 in the presence of 4 μg/mL of polybrene for 24 h, then replaced the virus medium with fresh growth medium and continued to culture the cells for 48 h. The cells were selected with 4 μg/mL of puromycin for 3 days. Then combined both half libraries cells and collected 2.5 × 10^7^ cells for genomic DNA isolation. Next-generation sequencing was performed on the Illumina HiSeq 2500 platform in Deep Sequencing Facility of TU Dresden. The raw FASTQ files were analyzed with MAGeCK-VISPR.

Additional methods are available in the Supplements.

## Supplementary information

Supplementary Figures

Supplementary Figure Legends

Supplementary Methods

Supplementary Table 1

Supplementary Table 2

## Data Availability

RNA-sequencing data have been deposited to GEO dataset GSE154429.
