# Supplementary material for: The role of miR-200b/c in balancing EMT and proliferation revealed by an activity reporter
Source: Oncogene. 2021 Mar 2;40(12):2309–22. doi: 10.1038/s41388-021-01708-6 (PMC7994202; doi:10.1038/s41388-021-01708-6)
Supplement: Supplementary file 1 — Supplementary Figures [file 41388_2021_1708_MOESM1_ESM.pdf]

Supplementary Figure 1

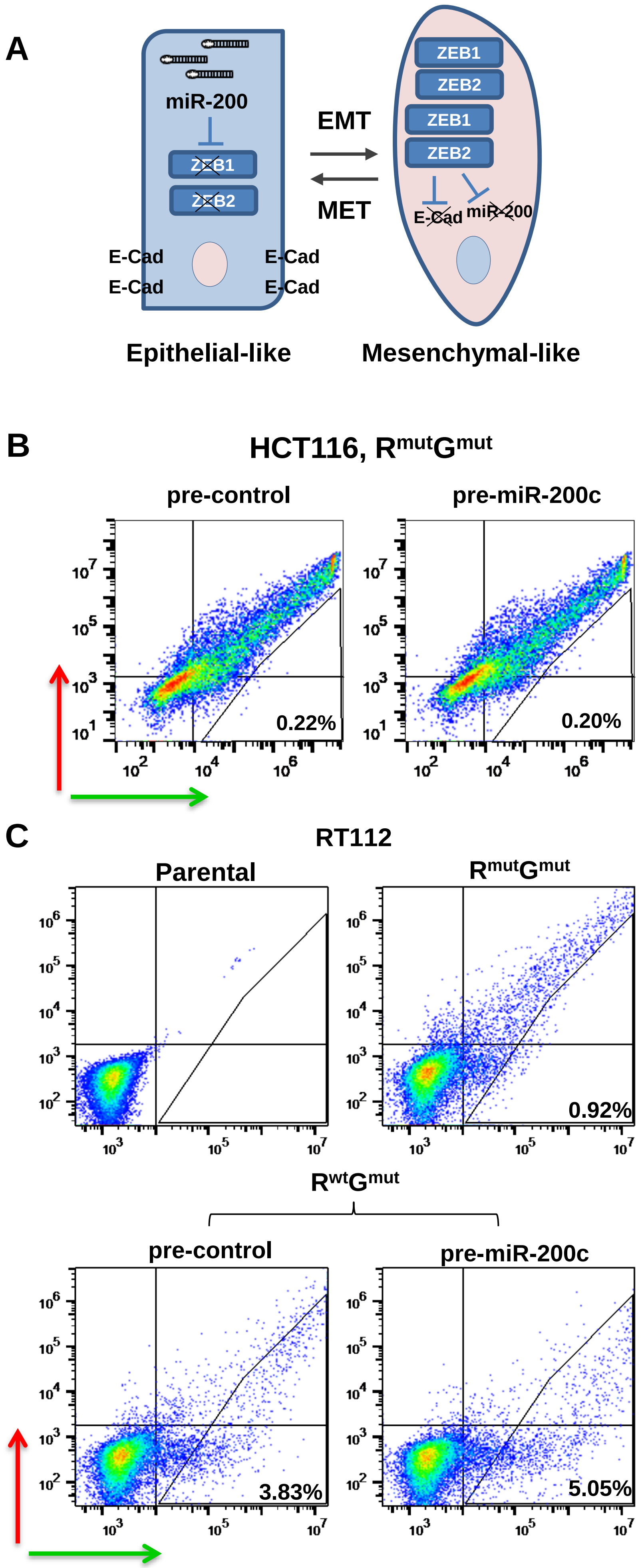

Supplementary Figure 2

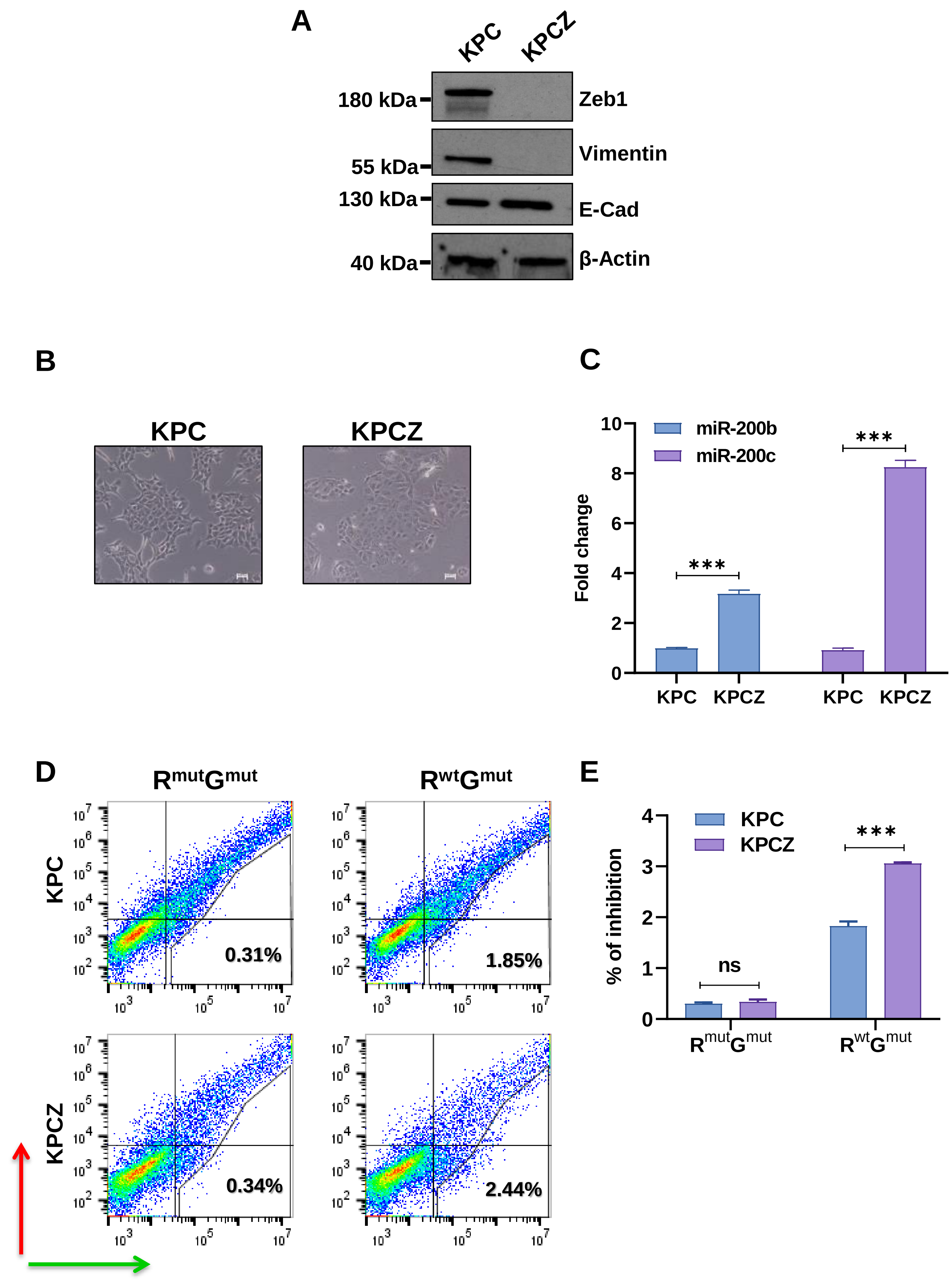

Supplementary Figure 3

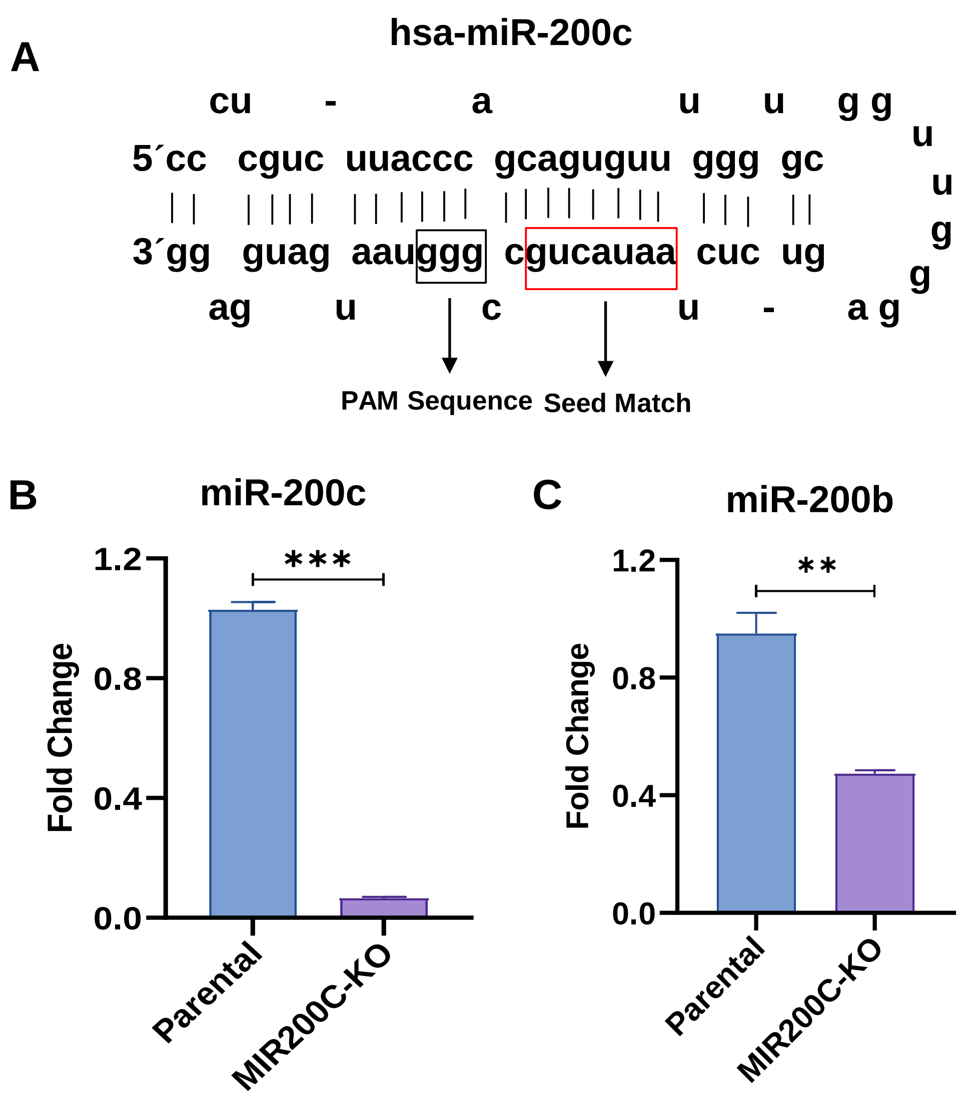

D

MIR200C

```
CloneIII     CCTGAAGCTGCCTGACCCAAGGTGGGCGGGCTGGGCGGGGGCCCTCGTCTTACCCAGCAG     119
CloneI       CCTGAAGCTGCCTGACCCAAGGTGGGCGGGCTGGGCGGGGGCCCTCGTCTTACCCAGCAG     120
CloneII      CCTGAAGCTGCCTGACCCAAGGTGGGCGGGCTGGGCGGGGGCCCTCGTCTTACCCAGCAG     119
CloneIV      CCTGAAGCTGCCTGACCCAAGGTGGGCGGGCTGGGCGGGGGCCCTCGTCTTACCCAGCAG     119
miR200c      -----------------------------------------CCCTCGTCTTACCCAGCAG     19
                                                      *******************

CloneIII     TGTTTGGGTGCTGATGATAGACGCTCATGTCTGTGTGTCAACGACGTCCATCCCCGTCCG     179
CloneI       TGTTTGGGTGCGGTTGGGATCTTGTTGGCTGGCTGCGACTATGGTACACGATGGTCTCCA     180
CloneII      TGTTTGGGTGCGGTTGGGAGTCTCTAATACTTGCGGGGAAAATATAGACGCCTGTGTCTG     179
CloneIV      TGTTTGGGTGCGGTTGGGAGTCTCTAATACTTGCCGGGTAATGATGGAGGCCCCTGTCCC     179
miR200c      TGTTTGGGTGCGGTTGGGAGTCTCTAATACTGCCGGGTAATGATGGAGG-----------     68
             *********** * **  *     *           *
```

MIR200B

```
CloneIV      CCTCAGCCGGGCGGCCCCCGGACCCAGCTCGGGCAGCCGTGGCCATCTTACTGGGCAGCA     180
CloneIII     CCTCAGCCGGGCGGCCCCCGGACCCAGCTCGGGCAGCCGTGGCCATCTTACTGGGCAGCA     178
CloneII      CCTCAGCCGGGCGGCCCCCGGACCCAGCTCGGGCAGCCGTGGCCATCTTACTGGGCAGCA     179
CloneI       CCTCAGCCGGGCGGCCCCCGGACCCAGCTCGGGCAGCCGTGGCCATCTTACTGGGCAGCA     179
miR2oob      -----------------------CCAGCTCGGGCAGCCGTGGCCATCTTACTGGGCAGCA     37
                                    *************************************

CloneIV      TTGGATGGAGTCAGGTCTCTAATACTGCCTGGTAATGATGACGGCGGAGCCCTGCACGCA     240
CloneIII     TTGGATGGAGTCAGGTCTCTAATACTGCCTGGTAATGATGACGGCGGAGCCCTGCACGCA     238
CloneII      TTGGATGGAGTCAGGTCTCTAATACTGCCTGGTAATGATGACGGCGGAGCCCTGCACGCA     239
CloneI       TTGGATGGAGTCAGGTCTCTAATACTGCCTGGTAATGATGACGGCGGAGCCCTGCACGCA     239
miR2oob      TTGGATGGAGTCAGGTCTCTAATACTGCCTGGTAATGATGACGGCGGAGCCCTGCACG--     95
             **********************************************************
```

Supplementary Figure 4

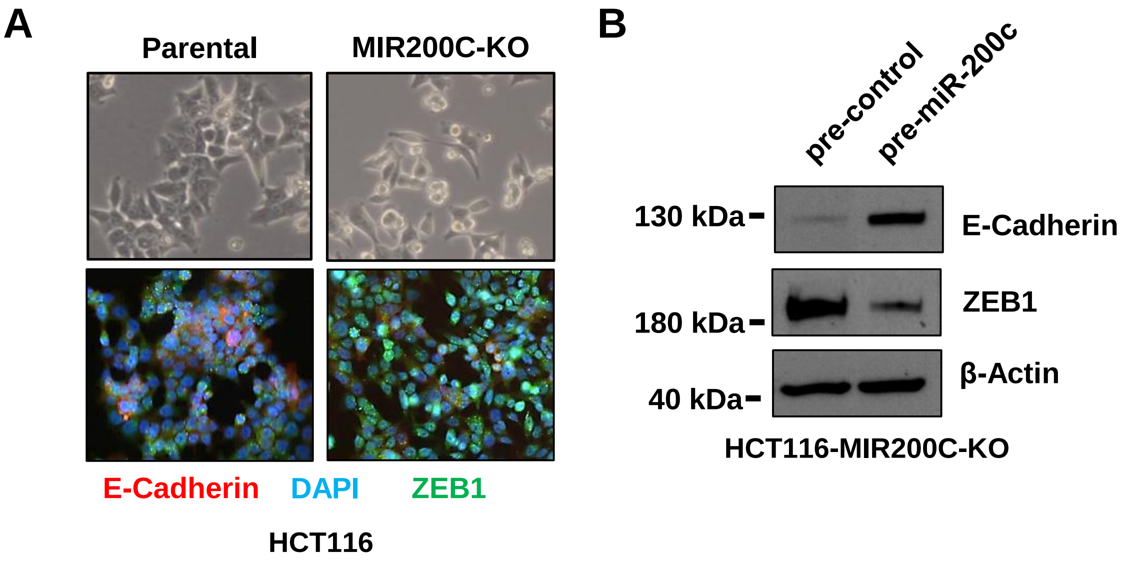

Supplementary Figure 5

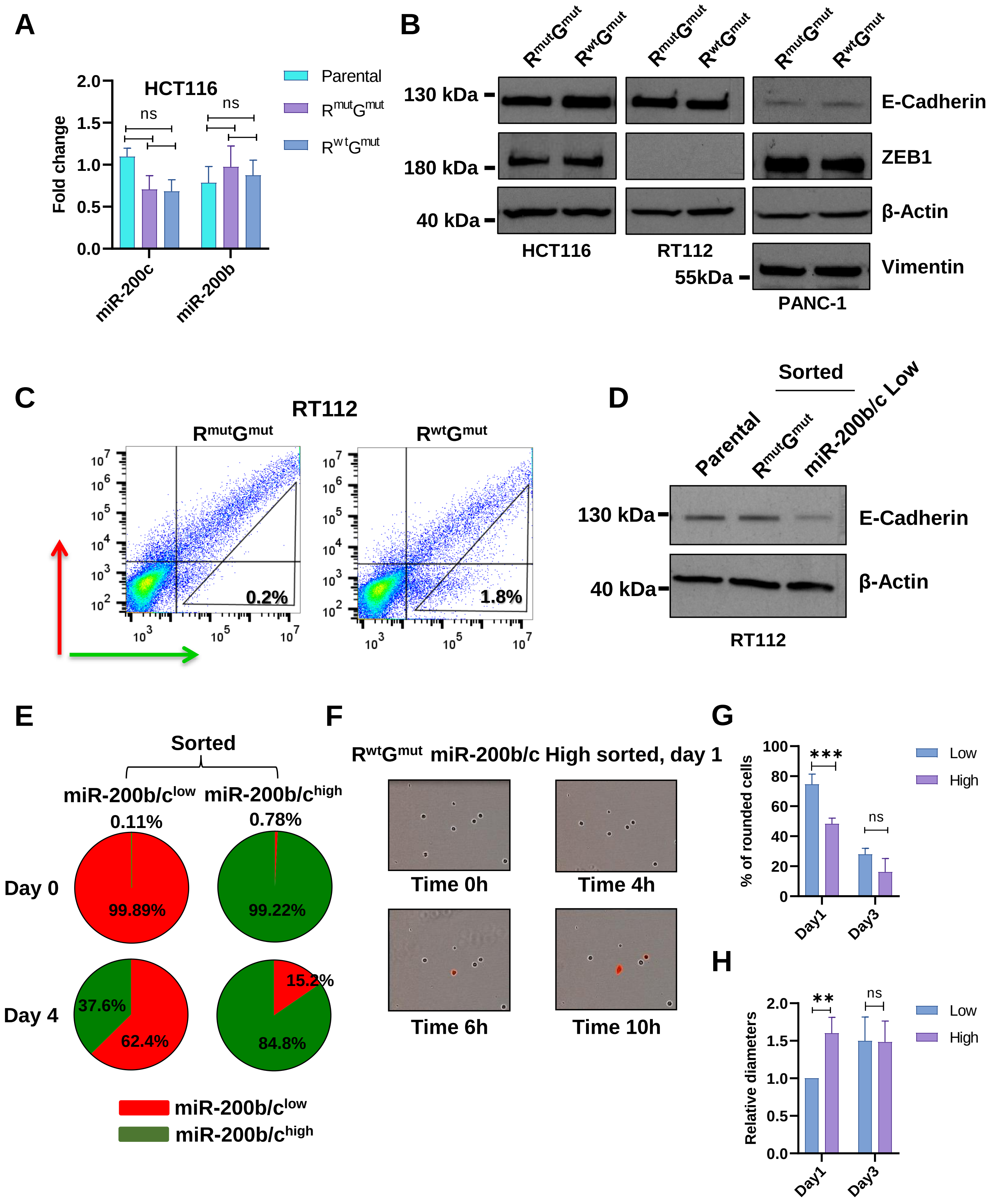

Supplementary Figure 6

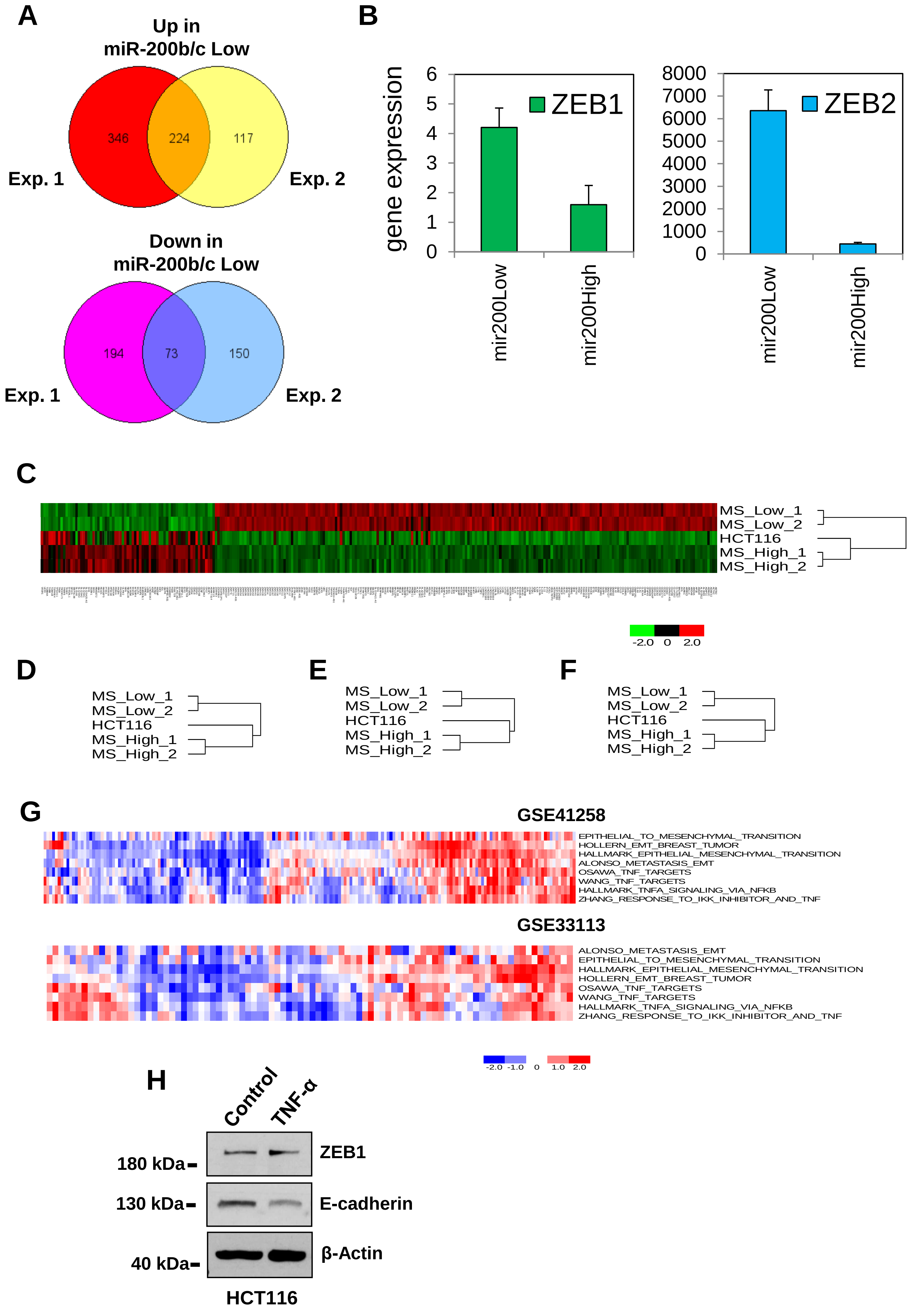

Supplementary Figure 7

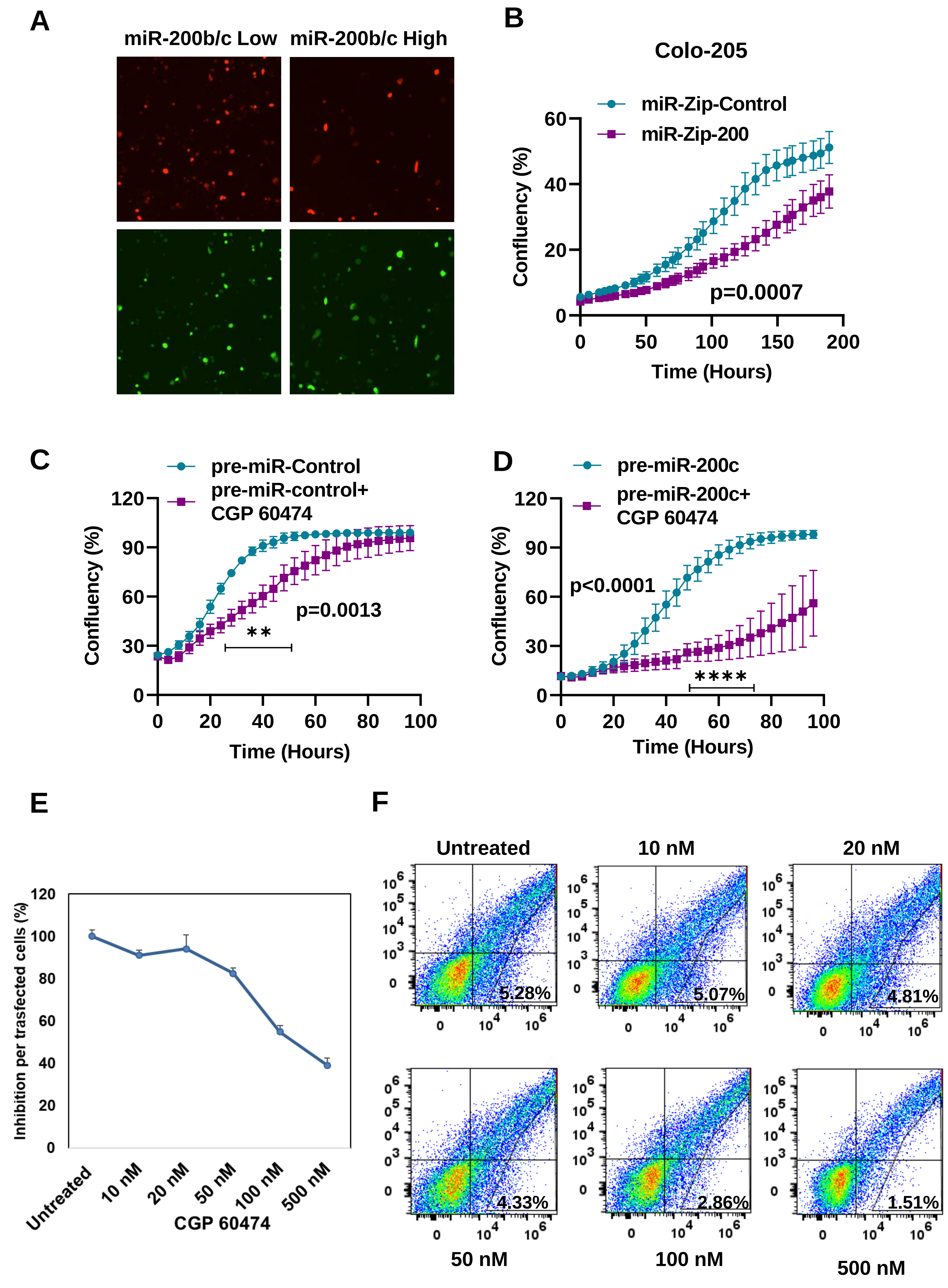

Supplementary Figure 8

A

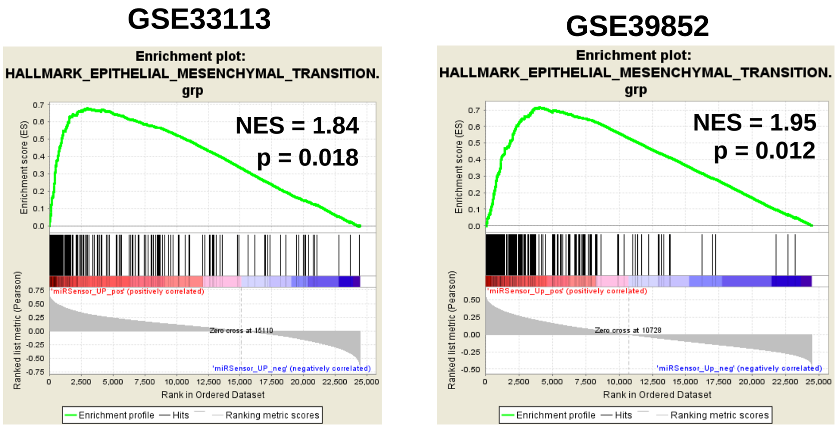

B

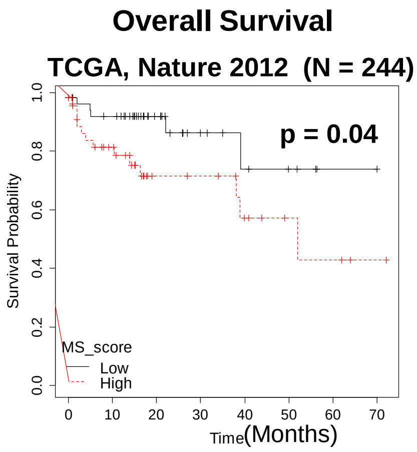

Supplementary Figure 9

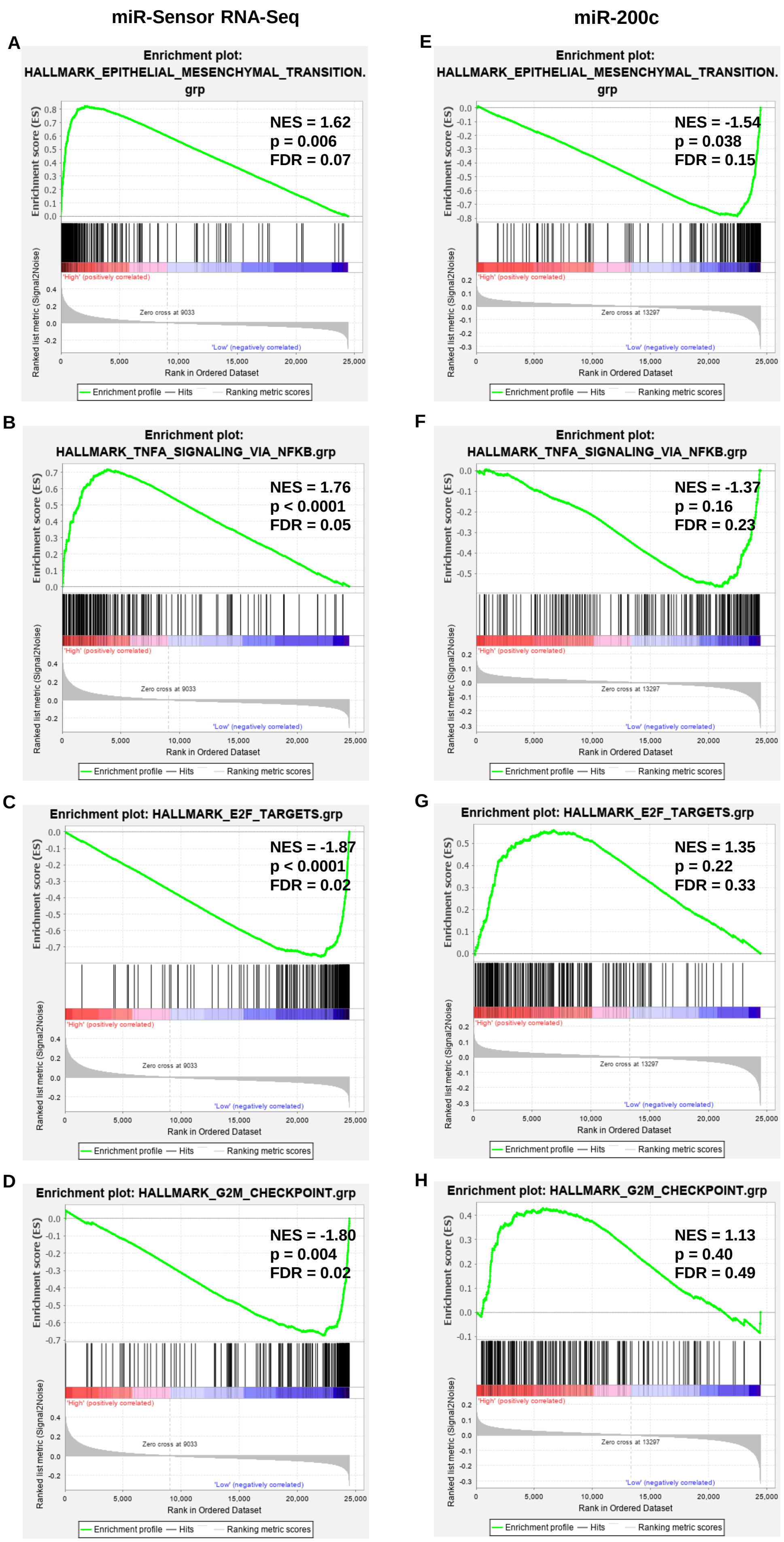

Supplementary Figure 10

A

	Label	TotalsgRNA	ZeroCounts	GiniIndex
1	hct-hab-c	119461	12264	0.21
2	hct-hab-h	119461	100644	0.88

Table 2: Summary of comparisons

2 Normalized read count distribution of all samples

The following figure shows the distribution of median-normalized read counts in all samples.

B

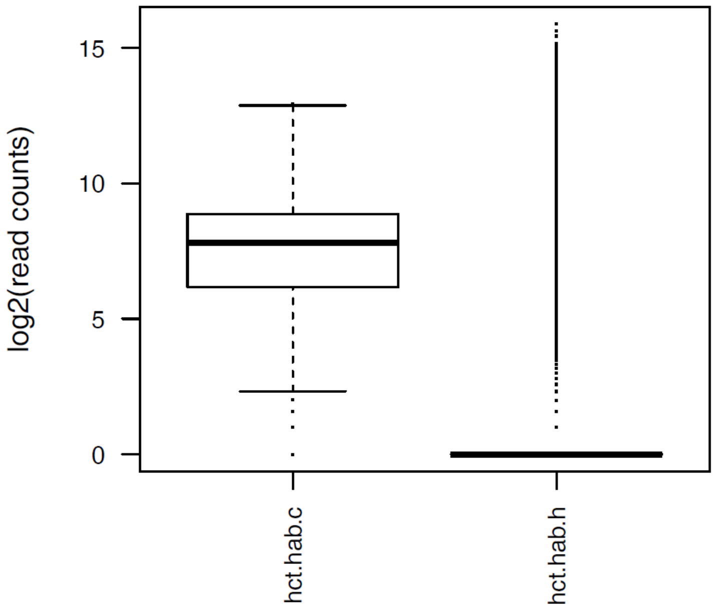

The following figure shows the histogram of median-normalized read counts in all samples.

Supplementary Figure 11

A

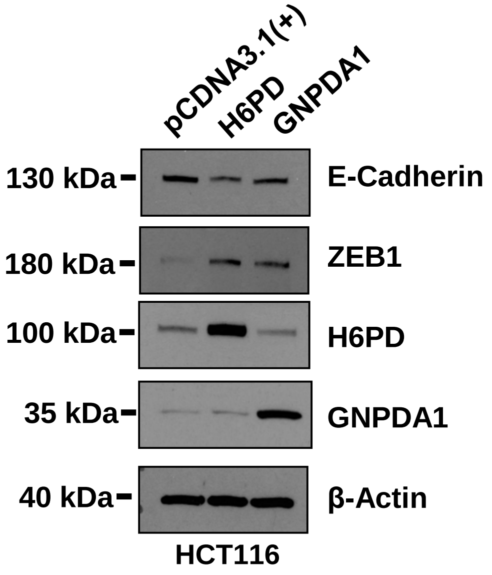

B

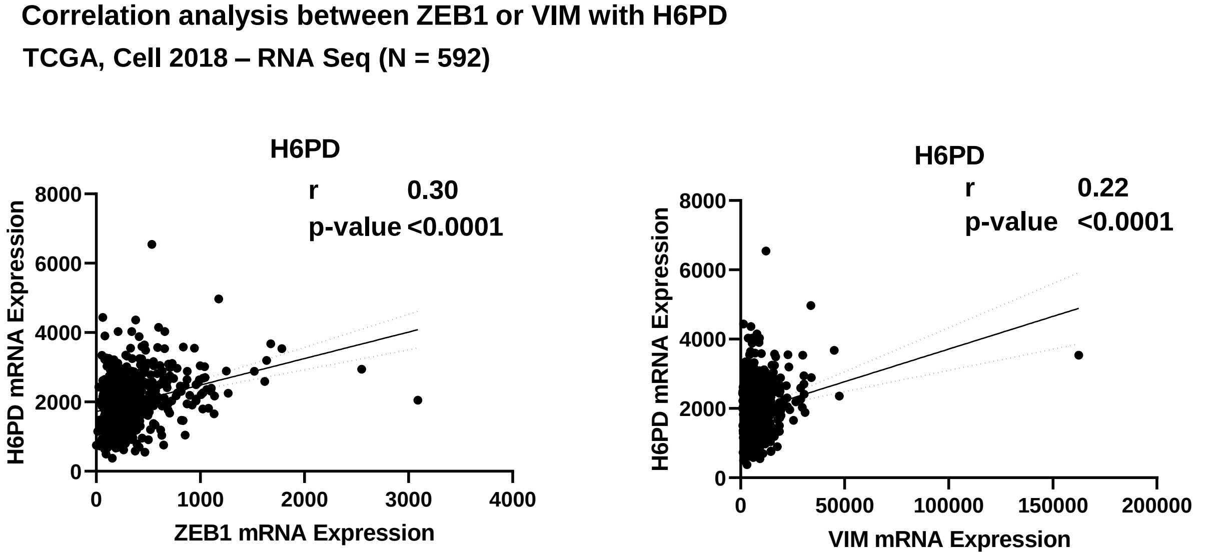

Supplementary Figure 12

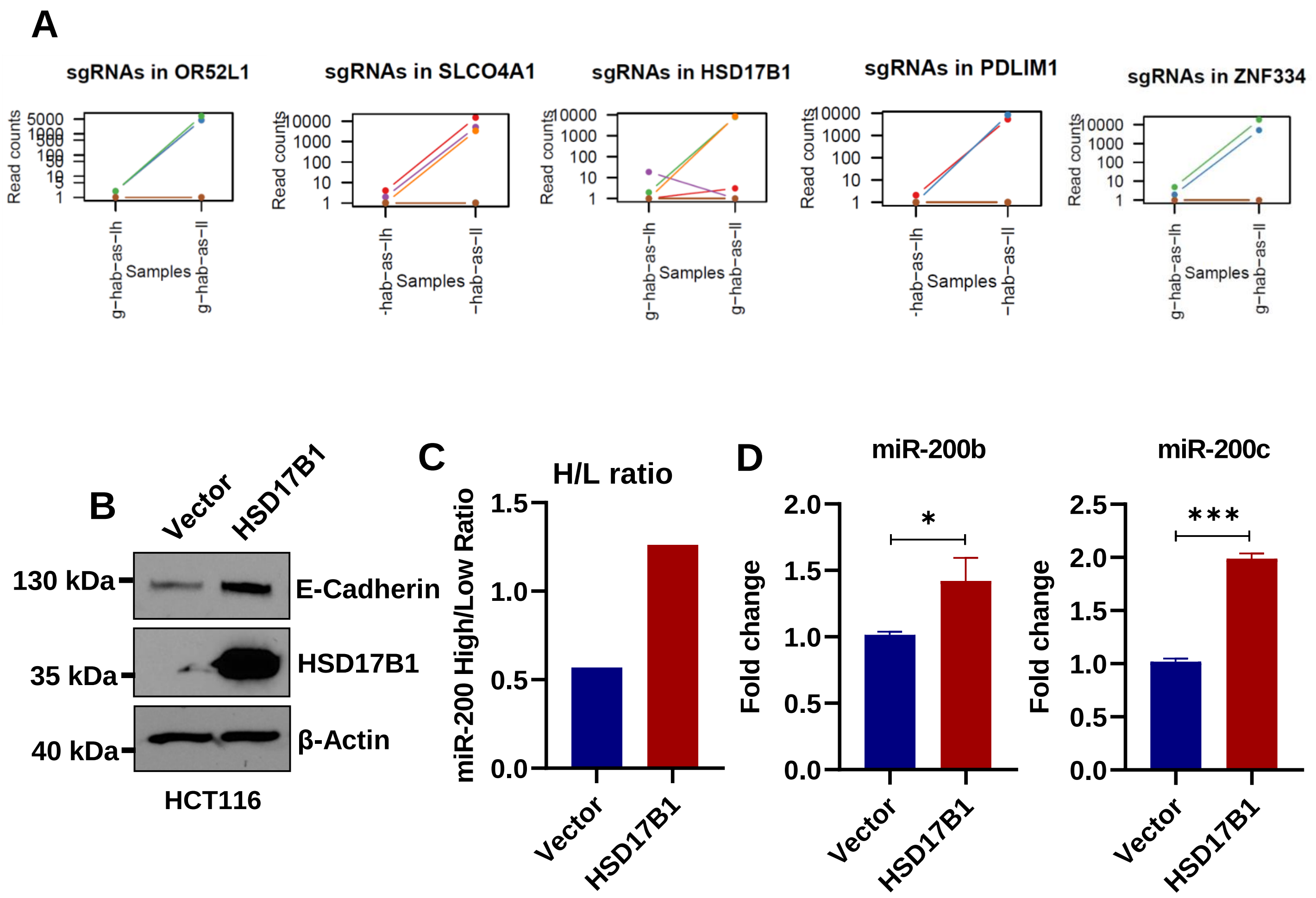

Supplementary Figure 13

A

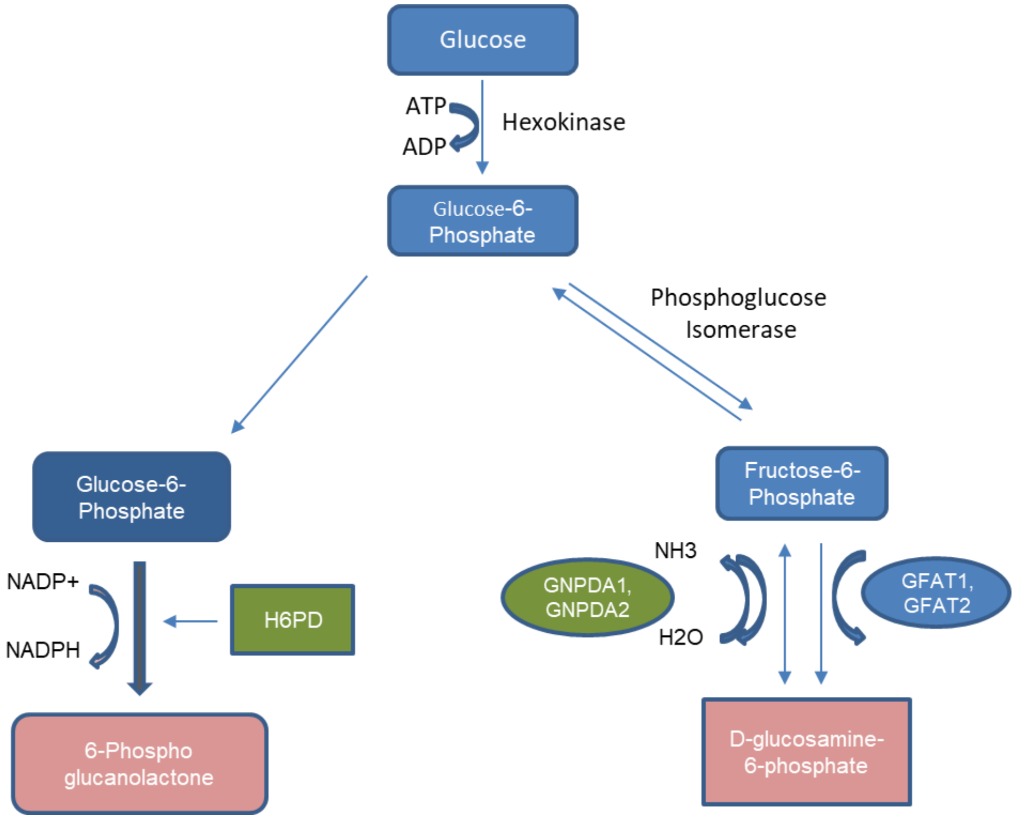

B

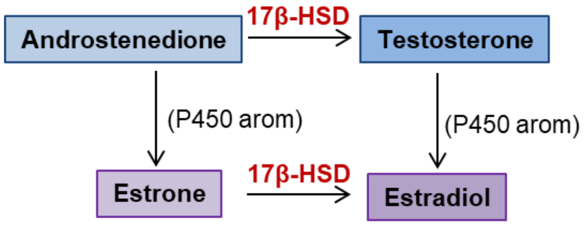

Supplementary Table 1A

Genes Up regulated in miR-200b/c Low				
ABTB2	EGR2	LOC100270804	SBDS	ZNF430
ACTA2	EID2	LOC100272217	SELK	ZNF431
ADAM32	ETS1	LOC100289230	SERPINE1	ZNF436
ADRA2C	EXOC8	LOC100506844	SIK1	ZNF441
AGAP11	FAM179B	LOC101928841	SLC25A21-AS1	ZNF554
ALOXE3	FAM200A	LOC644656	SMAP1	ZNF555
ALS2CR12	FAM214A	LOC646903	SMPD1	ZNF594
ANKAR	FAM46B	LRRC48	SNAI2	ZNF596
APOE	FLJ31104	LSMEM1	SNAPC1	ZNF624
ARID4A	FOXA1	LTBP2	STAM-AS1	ZNF654
ARID4B	FRS2	LTN1	SYNJ1	ZNF669
ARL14	GATA3	LVRN	TAF7L	ZNF674-AS1
ARRDC3	GEM	MAFF	TBC1D22B	ZNF721
AURKC	GNRH1	MAGEB3	TBX3	ZNF79
AVIL	GPATCH2	MAP3K8	TEF	ZNF821
BHLHE41	GPR3	MBIP	TFE3	ZNF833P
C17orf97	HCFC2	MED26	THAP1	ZNF92
C18orf65	HDAC9	MEX3B	THUMPD3-AS1	ZSCAN12P1
C1orf162	HIST1H2BD	MGARP	TIGAR	ZSCAN5A
C3orf38	HIST1H4E	MGC16142	TIGD4	ZSWIM3
CAPN10-AS1	HIST2H2BE	MOK	TMED6	ZNF430
CASS4	HOTAIRM1	MSH4	TMEM44-AS1	ZNF431
CCDC13	HSD17B7P2	MTTP	TNFAIP3	ZNF436
CCDC173	ICA1L	MURC	TPI1P2	ZNF441
CCDC30	IFIT2	MXD1	TRIM52	ZNF554
CCDC62	IL11	MYPN	TYW1B	ZNF555
CDC14A	IL23A	N4BP2L1	UBC	ZNF594
CDKN2AIP	IL31RA	NPR3	UCP3	ZNF596
CDKN2B	IL6R	NR4A3	VWCE	ZNF624
CFAP43	IQUB	OVGP1	WDR47	ZNF654
CHRM4	ITGA1	PARD6B	WDR78	ZNF669
CLEC4A	ITGAM	PCF11	YAF2	ZNF674-AS1
CLK1	JUN	PDLIM2	YOD1	ZNF721
CLK4	KBTBD8	PER2	ZBTB49	ZNF79
CNEP1R1	KDM4D	PIP5K1A	ZBTB6	ZNF821
COQ10B	KIAA0825	PLA2G15	ZEB1	ZNF833P
CPEB2	KLF10	PLA2G4C	ZEB1-AS1	ZNF92
CRY1	KLLN	PLK2	ZEB2	ZSCAN12P1
CSRNP1	KRCC1	PRDM1	ZFPM2-AS1	ZSCAN5A
CTC-338M12.4	KRTAP5-AS1	PRDM11	ZKSCAN4	ZSWIM3
CXCL8	LAMB3	PYGM	ZNF112	ZNF833P
CYLD	LATS2	RAET1L	ZNF14	ZNF92
DDR2	LCA5	RASGRF1	ZNF192P1	ZSCAN12P1
DKFZP434I0714	LCA5L	RBM24	ZNF211	ZSCAN5A
DLX2	LEKR1	RELB	ZNF23	ZSWIM3
DNAH10	LGALSL	RFPL3S	ZNF230	
DNAJB2	LINC00659	RPL32P3	ZNF250	
DNAJC27	LINC01004	RRN3P1	ZNF267	
DRC1	LINC01024	RYBP	ZNF333	
DUSP8	LINC01089	SAA2	ZNF34	
DZANK1	LINC01588	SAXO2	ZNF425	

Supplementary Table 1B

Genes Down regulated in miR-200b/c Low	
ADAM19	SDC3
ADAMTS14	SGSM1
AKAP1	SIX3
C14orf1	SLC25A25-AS1
C14orf80	SLC29A1
CA12	SLC29A2
CALHM2	SLC29A3
CCNF	SLC5A3
CDC25B	SMPDL3B
CDCA3	SPC25
CDK18	ST6GAL1
CSPG4	TEAD2
CTDSPL	THEM6
DNMT3B	TMEM171
E2F2	TNS4
EHBP1L1	TP53I11
EIF4EBP2	TSPAN14
ELFN1-AS1	TUBA1B
FAM72B	TUBB
FGD3	TUBB4A
FOXRED2	UCP2
GABPB1-AS1	VASH1
GMPR	
ID1	
IGF2	
INCENP	
KCNQ2	
KSR2	
LINC00342	
LRP8	
LRRC45	
MARCKSL1	
MEGF6	
MLPH	
MTERF4	
MUC3A	
MYADM	
NEURL1B	
NXN	
OLFM2	
PACSIN3	
PAQR4	
PDE4A	
POLR3K	
PPM1M	
PRSS16	
PTMA	
RAPGEF5	
RCCD1	
RMI2	
RNASET2	

Supplementary Table 2

top 10 in miR200H	top 10 in miR200L
ZNF687	OR52L1
EIF2D	MED10
GNPDA1	HTN1
H6PD	SLCO4A1
LCN10	HSD17B1
BTBD9	SAMD13
CHRNA2	PDLIM1
F9	NPC1L1
TRIB3	TMEM81
PROS1	ZNF334
