# Supplementary material for: The role of miR-200b/c in balancing EMT and proliferation revealed by an activity reporter
Source: Oncogene. 2021 Mar 2;40(12):2309–22. doi: 10.1038/s41388-021-01708-6 (PMC7994202; doi:10.1038/s41388-021-01708-6)
Supplement: Supplementary file 2 — Supplementary Figure Legends [file 41388_2021_1708_MOESM2_ESM.docx]

**SUPPLEMENTARY LEGENDS:**

**Supplementary Fig. 1.** A) Schematics showing the role of miR-200 in the regulation of epithelial to mesenchymal transition (EMT) and the feedback loop with the E-Cadherin repressors ZEB1/2. B) FACS plots showing green and red fluorescence in HCT116 transfected with the R^mut^G^mut^ control plasmid in the presence of 30 nM pre-control or pre-miR-200c. C) FACS plots showing transfection of RT112 cells with R^mut^G^mut^ or R^wt^G^mut^ plasmids in the presence of pre-control or pre-miR-200c.

**Supplementary Fig. 2.** A) Western blots showing abrogation of Zeb1 and Vimentin and upregulation of E-Cadherin in KPCZ compared to KPC cells. B) Microscopic images of KPC (Pdx1-cre;Kras^LSL.G12D^/+;Tp53^LSL.R172H/+^) and KPCZ (Pdx1-cre;Kras^LSL.G12D^/+;Tp53^LSL.R172H/+^; ZEB^fl/fl^) cells. Scale bar is 50 µM. C) qPCR quantification of relative expression levels of miR-200b and miR-200c in KPC and KPCZ cells. D) FACS plots showing transfection of KPC and KPCZ cells with R^mut^G^mut^ or R^wt^G^mut^ plasmids. E) Bar graphs showing % of inhibition with miR-200b/c sensor in KPC and KPCZ cells, as in D). Points are average±SD. p-values are from student’s *t*-test. *<0.05, **<0.01, ***<0.001, ****<0.0001.

**Supplementary Fig. 3.** A) Structure of human miR-200c showing the protospacer adjacent motif (PAM) sequence adjacent to the miRNA-200c seed match. qPCR quantification of relative B) miR-200c and C) miR-200b levels upon CRISPR/Cas9 mediated knockout (KO) of miR-200c in HCT116 cells compared to parental cells. Points are average±SD. p-values are from student’s *t*-test. D) Sequence alignments of single cell clones obtained from HCT116-MIR200C-KO cells comparing with the sequences of miR-200c and miR-200b. Underlined are mutations compared to the reference sequences. *<0.05, **<0.01, ***<0.001, ****<0.0001.

**Supplementary Fig. 4**. A) Fluorescent microscopic images showing E-Cadherin (Red) and ZEB1 (Green) levels in HCT116 parental and MIR200C-KO HCT116 cells. Dapi (Blue) is used to stain the nucleus. B) Western blot quantification of E-Cadherin and ZEB1 protein levels in HCT116 MIR200C-KO cells transfected either with pre-miR-control or pre-miR-200c. β-Actin was used as loading control.

**Supplementary Fig. 5.** A) qPCR quantification of relative expression levels of miR-200b and miR-200c in HCT116 cells upon transfection with R^mut^G^mut^ or R^wt^G^mut^ plasmids. B) Western blots showing the levels of E-Cadherin and ZEB1 in HCT116 and RT112, and E-Cadherin, ZEB1 and Vimentin in PANC-1 cells transfected with either R^mut^G^mut^ or R^wt^G^mut^ plasmids. β-Actin was used as loading control. C) FACS plots showing the separation of RT112 cells based on their miR-200b/c levels by using the sensor (only miR-200b/c low cells could be grown and analysed). D) Western blots showing the levels of E-Cadherin and β-Actin (loading control) proteins in parental RT112 cells and in sensor sorted cells. E) Pie charts showing the % of cells with miR-200b/c low and high on day 0 (as measured after sorting) and day 4 re-plated after sorting, as evaluated by FACS analysis. F) Images taken with live cell fluorescence imager, showing the sorted miR-200b/c high HCT116 cells increasing red fluorescence. Bar graphs showing G) % of rounded cells and H) relative diameter of HCT116 miR-200b/c low and high cells at day1 and day3 after sorting.

**Supplementary Fig. 6.** A) Venn**-**diagram showing overlap between the gene lists obtained from the two independent RNA-sequencing of miR-200b/c high and low cells from the two sorting experiments (Exp.1 and 2). B) ZEB1 and ZEB2 relative expression levels from RNA sequencing obtained from the sensor sorted miR-200b/c high and low cells. Points are average±SD. Heat-map visualization of the sample based clustering of parental HCT116 with miR-200b/c based sorting of low and high cells for RNA-Seq gene signature (C), ATAC-Seq of miR-200b 3’-UTR region gene signatures of 6-mer (D), 7-mer (E) and 8-mer (F) seed regions. G) Heatmap visualization of EMT and TNF-alpha gene signature activity pattern in colorectal cancer samples**.** Shown are the z-score activity pattern of EMT-associated and TNF-alpha signatures in colorectal cancer gene expression profiles, GSE33113 (N=90) and GSE41258 (N=186), indicating co-regulated activity, ranging from a minimum correlation of 0.35 to a maximum of 0.75 with an adjusted p-value <0.01. H) Western blot quantification of ZEB1 and E-Cadherin protein levels with or without TNF-alpha treatment (30 ng/mL) in HCT116 cells.

**Supplementary Fig. 7.** A) Images taken with live cell fluorescence imager, showing the red and green fluorescence in sorted miR-200b/c low and high HCT116 cells from the Figure 3F. B) Graph showing the proliferation of COLO 205 cells stably expressing miR-Zip control or miR-Zip-200. Cell proliferation in HCT116 cells treated with C) pre-miR-control alone or pre-miR-control with Cdk inhibitor (CPG-60474, 20 nM) and D) pre-miR-200c alone or pre-miR-200c with Cdk inhibitor (20nM). E) Graph showing the % of inhibition in HCT116 cells transfected with the R^wt^G^mut^ plasmid in presence of the indicated concentrations of Cdk inhibitor CGP-60474. F) FACS plots showing the % of inhibition in HCT116 cells in presence or absence of increasing doses of CGP-60474. In B, C and D, the p-values are from two-way ANOVA and Sidak’s multiple test. Points are average±SD. *<0.05, **<0.01, ***<0.001, ****<0.0001.

**Supplementary Fig. 8.** A) Gene-set enrichment analysis of genes up-regulated in miR-200b/c low cells with hallmark EMT geneset in the indicated datasets using Pearson’s gene metric. B) Kaplan-Meier analysis of overall survival (TCGA, Nature 2012) in colorectal cancer patients based on the median value of MS score. p-values were calculated using log-rank test.

**Supplementary Fig. 9.** Gene-set enrichment analysis of hallmark genesets (EMT, G2M, E2F and TNFA) with the low and high categorized patient samples based on (A-D) MS score of RNA-seq genes obtained from the sensor-sorting experiment, and based on (E-H) miR-200c expression in the GSE81980 dataset. GSEA was performed with the ranking of genes with signal2noise metric.

**Supplementary Fig. 10.** A) Table showing the total gRNAs, the number of absent gRNA (zero counts) in control (hct-hab-c) and miR200b/c-high cells (hct-hab-h). B) Box-plot showing the distribution of read counts in control (hct-hab-c) and miR200b/c-high cells.

**Supplementary Fig. 11.** A) Western blot quantification of EMT markers ZEB1 and E-Cadherin, as well as H6PD and GNPDA1 in cells stably transfected with the cDNA clones overexpressing H6PD and GNPDA1, compared to the pCDNA3.1 vector control. β-Actin was used as loading control. B) Correlation analysis of H6PD mRNA with that of either ZEB1 or Vimentin, in TCGA RNA sequencing data of colorectal adenocarcinoma patients (n=592).

**Supplementary Fig. 12.** A) Graphs showing the read counts of the gRNAs corresponding to the indicated genes in sensor-sorted miR-200b/c high cells compared to low cells. B) Western quantification of E-Cadherin and HSD17B1 levels in HCT116 cells stably transfected with either vector (pCDNA3.1) or HSD17B1 cDNA plasmid. C) Graph showing the ratio of miRNA-200b/c high and low cells in cells as in (B) transfected with the R^wt^G^mut^ plasmid and analysed by FACS. D) qPCR analysis of miR-200b (left) and miR-200c (right) in HCT116 cells stably transfected with either vector (pCDNA3.1) or HSD17B1 cDNA plasmid. Points are average±SD. p-values are from students *t*-test. *<0.05, **<0.01, ***<0.001, ****<0.0001.

**Supplementary Fig. 13.** Scheme showing the metabolic functions of A) H6PD and GNPDA1 and B) HSD17B1.

**Supplementary Table 1.** A) Genes up- regulated in sensor-sorted miR-200b/c low HCT116 cells compared to miR-200b/c high cells B) Genes down- regulated in sensor-sorted miR-200b/c low HCT116 cells compared to miR-200b/c high cells. Identified through RNA-Sequencing.

**Supplementary Table 2.** Top 10 hits genes identified by the CRISPR/Cas9 screens of high and low miR-200b/c HCT116 cells.

.
