# Supplementary material for: The role of miR-200b/c in balancing EMT and proliferation revealed by an activity reporter
Source: Oncogene. 2021 Mar 2;40(12):2309–22. doi: 10.1038/s41388-021-01708-6 (PMC7994202; doi:10.1038/s41388-021-01708-6)
Supplement: Supplementary file 3 — Supplementary Methods [file 41388_2021_1708_MOESM3_ESM.docx]

**SUPPLEMENTARY METHODS**

**Generation of miR-200b/c sensor**

The cloning of the sensor plasmid was previously described (24). Briefly, DNA fragments of the ZEB2 3’UTR (from nt 782 to 1080, containing miR-200b/c seed numbers 4, 5 and 6) were linked to GFP (pAcGFP1-Hyg-C1) and DsRed (pDsRed-Express-C1) containing plasmids (from Clontech Laboratories). As inactive controls, 3’UTRs with mutated miR-200b/c seed matches (CAGTATT to CTGAAAT) were produced (DNA synthesis from IDT). Wild-type or mutated GFP-3’UTR or DsRed-3’UTR were cloned in tandem into CD510B-1 vector (System Biosciences) driven by two identical CMV promoters, see scheme in **Figure 1A**.

**Transfection**

Transfection of sensor plasmids were done using polyethyleneimine (PEI) mixing plasmid and PEI in 1:3 ratio in 0.9% NaCl. After 20 minutes of incubation, the mixture was added drop wise to the cells. Co-transfection of miRNA mimics (Ambion) and sensor plasmids was done with lipofectamine2000 or LTX (Life technologies). pCDNA3.1(+)-Hygro plasmids and empty vector control were purchased from Genscript and transfected with PEI. pCDNA3.1(+)-Hygro-transfected cells were selected and grown as stable cultures adding 250 µg/ml hygromycin in the culture media.

**Western Blot**

Cells were lysed in RIPA buffer and protein concentration was measured using BCA kit (Thermo Fisher). Protein lysates were resolved on 8-10% gels and transferred to PVDF membrane. Membranes were blocked in 5% milk in TBST for 1 hour and incubated with the primary antibodies overnight. Membranes were then washed with TBST and incubated with secondary antibodies for 1 hour. The membranes were developed with ECL reagent (Thermo Fisher) on to X-ray films using the chemiluminescence imager, AGFA CP100. Antibodies used are E-Cadherin (cat no. 14472), Vimentin (cat. no. 5741), β-Actin (HRP conjugated, cat. no. 12262) from Cell Signalling Technologies, ZEB1 (cat. no. HPA027524) from Sigma-Aldrich, H6PD (cat. no. MA525125) and GNPDA1 (cat. no. PA520693) from Thermo Fisher, HSD17B1 (cat. no. ab51045) from Abcam. Cas9 antibody (bD-20, cat. no. sc-392737) was purchased from Santa Cruz Biotechnology. Horseradish Peroxidase (HRP)-conjugated secondary antibodies (cat. no. 1071-05 and 4030-05) were from Southern Biotech. Quantification of band intensity was carried out by Image J software. Changes of more than 2-fold in ZEB1/E-Cadherin ratio in cells with stable overexpression compared to controls (Figure 4E) were considered significant.

**Proliferation assay**

Cells were seeded in a 96 well plates at very low density (5-10% confluency). Proliferation was measured by IncuCyte ZOOM live cell imaging system (Essen BioScience) scanning every 2-4 hours. The phase contrast images acquired by IncuCyte ZOOM were used to train the ZOOM software to develop processing definition to mask the cells. The mask was further utilized to identify the cells in the images and determined the surface area at each scanned time point. The final output was represented as confluency percentage which was indicative of proliferation (n=6 technical replicates for each biological repeat).

**Immunofluorescence**

Immunofluorescence staining of cells grown on cover slips was performed as follows: HCT116 cells were grown on a glass cover slip until they were about 90% confluent. Cells were washed with PBS and fixed with 70% pre-chilled ethanol for 20 minutes at room temperature. Cells were blocked in 3% BSA in PBS for an hour after two PBS washes, followed by overnight incubation in 250th dilution of ZEB1 or E-Cadherin (same Abs used for western blotting) in the blocking buffer. After the incubation, cells were washed with PBS and incubated with 250th dilution of secondary mouse or rabbit antibody in blocking medium for an hour. Cells were washed thrice with PBS and the cover slip was mounted on the glass slide with histology mounting medium (Fluoroshield with DAPI, Sigma). Slides were visualized and photographed using Leica DM5500B fluorescence microscope and merged using Leica Application Suite-X software.

**Luciferase assay**

Luciferase assay for NF-κB was done using NF-κB reporter kit (BPS Bioscience) according to manufacturer’s instructions. Briefly, 25,000 HCT116 cells were seeded in each well of 96 well plate. Next day 1 µL of reporter plasmid was transfected using Lipofectamine2000 (Life Technologies). After 48 h the cells were lysed and luminescence was measured with Dual-Glo Luciferase assay system (Promega) according to the manufacturer’s instructions. Both Firefly and Renilla luminescence was measured and Firefly/Renilla ratio is taken as NF-κB activity. Experiments were performed at least twice independently (biological replicates), each time with n=6 technical replicates.

**qPCR**

Total RNA was extracted using miRNeasy kit (Qiagen) and 45 ng of RNA was retro-transcribed using TaqMan MicroRNA reverse transcription kit with the respective primers of miRNAs from TaqMan-microRNA assays (Applied Biosystems). qPCR was performed using probes for the respective miRNAs and Universal Mastermix (Applied Biosystems). RNU6B was used as internal control. For mRNAs, random hexamers primers and Tetro cDNA synthesis kit (Bioline) were used for cDNA synthesis and GAPDH was used as internal control. All gene expression assays were performed with TaqMan probes of the respective genes (Applied Biosystem). Quantifications were done by Applied Biosystems 7300 Real Time PCR system and fold change calculated using the ΔΔCt method. Experiments were performed at least twice independently (biological replicates), each time with n=3 technical replicates.

**Lentiviral Transduction**

Lentiviral particles were generated by transfecting HEK-293T cells with 8 μg of expression vectors (MIR200 CRISPR V2, miR-Zip control, miR-Zip-200c) and 2 μg each of packaging vectors (pMDL, pVsVg and pRevRes) in complex with 24 μg PEI (Polysciences) in 0.9% NaCl. Viral titer was allowed to concentrate for 48 hours, after which supernatant was collected, centrifuged at 400g for 5 minutes and passed through 0.22 μm syringe filter. For transduction, cells were seeded in 6-well plate (100,000 to 150,000) and infected in the presence of 8 μg/ml polybrene (Sigma). Cells were selected in medium containing 3 μg/ml puromycin (Sigma) and cultured in 1 μg/ml puromycin. MIR200 CRISPR V2 was custom-made and purchased from Genscript (sequence of the gRNA is TGGGAGTCTCTAATACTGCC). miR-Zip control and miR-Zip-200c plasmids were purchased from System Biosciences.

**RNA sequencing**

Total RNA was extracted using miRNeasy kit (Qiagen) following the manufacturer’s instructions. RNA-Seq libraries were constructed using the TruSeq sample Prep Kit V2 (Illumina). Briefly, 1-2 μg of purified RNA was poly-A selected and fragmented with fragmentation enzyme. After first and second strand synthesis from a template of poly-A selected/fragmented RNA, other procedures from end-repair to PCR amplification were done according to library construction steps. Libraries were purified and validated for appropriate size on a 2100 Bioanalyzer High Sensitivity DNA chip (Agilent Technologies.) The DNA library was quantified using Qubit and normalized to 4 nM before pooling. Libraries were pooled in an equimolar fashion and diluted to 10 pM. Library pools were clustered and run on Nextseq500 platform with paired end reads of 75 bases, according to the manufacturer’s recommended protocol (Illumina). Raw reads passing the Illumina RTA quality filter were pre-processed using FASTQC for sequencing base quality control. Sequence reads were mapped to UCSC human genome build using TopHat and differential gene expression determined using Cufflinks 2.1.1 and Cuffdiff2.1.1 as implemented in Base-Space.

**CRISPR/Cas9 library lentiviral generation and viral transduction**

Lentivirus was generated with HEK293TN cells in T175 flasks. Briefly, 9.2 µg pMDLg/pRRE plasmid (Addgene, #12251), 4.6 µg pRSV-REV plasmid (Addgene #12253), 4.6 µg pMD2.G plasmid (Addgene #12259) and 13.8 µg of Human CRISPR Knockout Pooled Library (GeCKO v2) (Addgene # 1000000049) part A or part B were combined with Lipofectamine 3000 (Thermo Fisher Scientific # L3000015) according to the manufacturer’s protocol, and transfected the cells with the mixture. The virus medium was collected 24h after transfection and centrifuged at 2000 rmp for 10 minutes to remove cells and debris. Then the supernatant was filtered with a 0.45 μM pore filter and frozen at -80°C. HCT116-Cas9 cells was transduced with serial dilutions of a virus to find the MOI of ~0.3.

**Genomic DNA isolation and PCR amplification**

Genomic DNA was extracted with NucleoSpin® Blood XL (Machery Nagel # 740950.50) according to the manufacturer’s protocol. The first round PCR of Next Generation Sequence (NGS) is performed with 26 separate 100-μL redundant reactions, each containing 5 μg of DNA, 50 μL Q5® Hot Start High-Fidelity 2X Master Mix (NEB # M0494L), and 5 μL of a 10 μM solution of each primer (P5 and P7). The PCR amplification program was as follows: step 1, 98°C for 30s; step 2, 98°C for 10s; step 3, 62°C for 30s; and step 4, 72°C for 30s; steps 2-4 is repeated for 25 times, step 5, 72°C for 2 min. Then the PCR production was sent to NGS.

Primer P5:

ACACTCTTTCCCTACACGACGCTCTTCCGATCTNNNNNTCTTGTGGAAAGGACGAAACACCG

Primer P7:

GTGACTGGAGTTCAGACGTGTGCTCTTCCGATCTTCTACTATTCTTTCCCCTGCACTGT

**Survival analysis**

Gene expression profiles of colorectal cancer patient samples were obtained as normalized values from GEO (GSE39582 and GSE33113) and mRNA z-score values for TCGA profile (TCGA, Nature 2012) from cbioportal platform. We generated a score by taking the difference of averaged z-score of up-regulated and down-regulated genes identified from RNA sequencing of FACS sorted high and low population of miR-200b/c sensor expressing cells and named it as MS (miRNA-Sensor) score. Samples were then categorized as MS-low and MS-high based on the median value to generate survival curve using Kaplan-Meier estimate. Significance between the two groups was assessed using log-rank test in R software.

**Gene Set Enrichment Analysis**

Gene set enrichment analysis (GSEA) was performed using GSEA 4.0.3 software for the association of miR sensor differentially regulated genes with ‘Hallmark EMT gene set’ in a patient gene expression profile obtained from GEO database (GSE39852 and GSE33113) and cbioportal platform for TCGA profile. miR sensor regulated genes score was provided as continuous label of averaged z-score values and calculated the gene ranks using Pearson ranked gene metric.

**Gene signature activation and associational analysis**

EMT, TNF-α, G2M and E2F related gene signatures were obtained from MSigDb v7.1 and assessed their gene signature activity using z-score method in colorectal patient mRNA expression profiles (GSE33113 and GSE41258). When up- and down-regulated gene signatures information were available for the study, a difference of z-score between up- and down-regulated gene signatures was carried out. With the z-score activity of the signatures across the colorectal samples, correlation analysis was performed in R using cor.mat function to assess the significance of association and plotted the graph using GraphPad Prism 8. For the sensitivity analysis of miR-sensor RNA seq genes, the difference between up- and down-regulated genes z-score activity was used to categorize the samples into low and high patient sample groups based on the median value in the colorectal cancer patient expression profile (GSE81980). miR-200c expression for the matched mRNA expression profile was obtained from GSE81981 to categorize the samples into low and high based on the median expression for the enrichment of Hallmark genesets.

**Cluster analysis**

FPKM values of RNA Sequencing expression profile from low and high sorted cells based on miR-sensor were converted to TPM values. TPM values of parental HCT116 was obtained from Broad Institute Cancer Cell Line Encyclopedia (CCLE). Sample based clustering was performed in dChip software with 1-correlation distance metric and using average linkage method for the miR-sensor gene signature and seed regions of miR-200b 3’-UTR ATAC sequencing gene signatures, and visualized as heat map.

**Extracellular flux assays**

OCAR measurement was determined using the Mito Stress Test (Agilent kit 103015-100) with XFe96 Extracellular Flux Analyzer (Seahorse Bioscience/Agilent Technologies). Cells were seeded at a density of 25,000 cells/well. One hour before the measurement, culture media was replaced with XF base medium (Agilent 103334-100) supplemented with 10 mM glucose (Sigma), 2 mM L-glutamine and 1 mM sodium pyruvate adjusted to pH 7.4 and cells were incubated at 37°C in a CO_2_-free atmosphere for 45-60 minutes. 1.5 μM oligomycin, 2 μM FCCP, 1 μM rotenone and 1 μM antimycin were sequentially injected at regular intervals. Basal oxygen consumption rate (OCR, indicator for mitochondrial respiration) was measured. Experiments were performed twice independently (biological replicates), each time with n=5 technical replicates.

**In vivo experiments**

NSG mice (from JAX) were used as experimental model to study the metastatization of miR200b/c high/low cells. After sorting, 50,000 cells were counted, re-suspended in 100 μl PBS and injected in the tail vein of 9 weeks old female NSG mice, n=3 per group. Sample size was chosen as previously (Siddiqui et al 2019 *Cell Death and Differentiation*). No randomization or blinding were used. After 5 weeks, mice were euthanized by cervical dislocation and macroscopic metastatic lesions were counted from isolated livers and kidneys. In vivo experiments were performed by skilled experimenters trained according to FELASA guidelines. Animal protocols were approved by the Institutional Animal Care and Use Committee of the Regierung von Unterfranken.

**Statistical analysis**

Unless otherwise specified, each *in vitro experiment* was performed with n=3 technical replicates, and repeated 3 times independently (biological repeats), and representative results are shown. Statistical tests (all two-sided) were performed with the GraphPad software v.8 comparing groups of different conditions with replicates (all assumed to have same variance). In all tests, the statistical significance was set at p≤0.05 (in the figures * indicates p<0.05, ** p<0.01, *** p<0.001).
